# Sleep-Dependent Reactivation of Ensembles in Motor Cortex Promotes Skill Consolidation

**DOI:** 10.1371/journal.pbio.1002263

**Published:** 2015-09-18

**Authors:** Dhakshin S. Ramanathan, Tanuj Gulati, Karunesh Ganguly

**Affiliations:** 1 Neurology and Rehabilitation Service, San Francisco VA Medical Center, San Francisco, California, United States of America; 2 Psychiatry Service, San Francisco VA Medical Center, San Francisco, California, United States of America; 3 Department of Psychiatry, University of California, San Francisco, San Francisco, California, United States of America; 4 Department of Neurology, University of California, San Francisco, San Francisco, California, United States of America; University of Minnesota, UNITED STATES

## Abstract

Despite many prior studies demonstrating offline behavioral gains in motor skills after sleep, the underlying neural mechanisms remain poorly understood. To investigate the neurophysiological basis for offline gains, we performed single-unit recordings in motor cortex as rats learned a skilled upper-limb task. We found that sleep improved movement speed with preservation of accuracy. These offline improvements were linked to both replay of task-related ensembles during non-rapid eye movement (NREM) sleep and temporal shifts that more tightly bound motor cortical ensembles to movements; such offline gains and temporal shifts were not evident with sleep restriction. Interestingly, replay was linked to the coincidence of slow-wave events and bursts of spindle activity. Neurons that experienced the most consistent replay also underwent the most significant temporal shift and binding to the motor task. Significantly, replay and the associated performance gains after sleep only occurred when animals first learned the skill; continued practice during later stages of learning (i.e., after motor kinematics had stabilized) did not show evidence of replay. Our results highlight how replay of synchronous neural activity during sleep mediates large-scale neural plasticity and stabilizes kinematics during early motor learning.

## Abstract 2

During non-REM sleep in rats, consolidation and offline improvements of a recently learned motor skill are linked to synchronous reactivation of task-related neural ensembles.

## Author Summary

Sleep has been shown to help in consolidating learned motor tasks. In other words, sleep can induce “offline” gains in a new motor skill even in the absence of further training. However, how sleep induces this change has not been clearly identified. One hypothesis is that consolidation of memories during sleep occurs by “reactivation” of neurons engaged during learning. In this study, we tested this hypothesis by recording populations of neurons in the motor cortex of rats while they learned a new motor skill and during sleep both before and after the training session. We found that subsets of task-relevant neurons formed highly synchronized ensembles during learning. Interestingly, these same neural ensembles were reactivated during subsequent sleep blocks, and the degree of reactivation was correlated with several metrics of motor memory consolidation. Specifically, after sleep, the speed at which animals performed the task while maintaining accuracy was increased, and the activity of the neuronal assembles were more tightly bound to motor action. Further analyses showed that reactivation events occurred episodically and in conjunction with spindle-oscillations—common bursts of brain activity seen during sleep. This observation is consistent with previous findings in humans that spindle-oscillations correlate with consolidation of learned tasks. Our study thus provides insight into the neuronal network mechanism supporting consolidation of motor memory during sleep and may lead to novel interventions that can enhance skill learning in both healthy and injured nervous systems.

## Introduction

The cardinal features of motor skill learning are enhanced speed and automaticity of motor execution with preserved accuracy [1–3]. Motor learning is known to progress through a series of stages: an early stage accompanied by rapid improvements in accuracy with continued variability of movement kinematics, followed by consolidation of these processes and transition to a later stage of learning, in which kinematics are largely stabilized but slow improvements in accuracy continue to occur [4–6]. The underlying neural basis by which kinematics become stabilized during early motor learning is not well understood. Human studies suggest that non-rapid eye movement (NREM) sleep is essential for this consolidation and, in addition, results in additional gains in skilled motor performance [7–14]. Even brief naps during the day can mediate these “offline” motor improvements [11,15,16], including faster movements and reduced variability in timing.

There is evidence that NREM sleep promotes offline gains. Prior studies have described a relationship between motor learning, local slow-wave oscillations [17], the expression of immediate-early plasticity-related genes [18] and stabilization of dendritic spines [19]. However, how large-scale patterns of neural activity drive plasticity of specific motor circuits to result in enhanced motor performance is unknown. Based primarily on studies of single-unit activity conducted during hippocampal-dependent behaviors [9,13,16,17,20–25], we hypothesized that reactivations of task-related emergent neural firing during NREM sleep may be related to subsequent neural plasticity and associated offline behavioral gains. This hypothesis is largely consistent with theoretical models for how NREM sleep promotes learning more generally [20–22,26–29], but to our knowledge, there is little experimental support of this during procedural memory formation.

## Results

### Experiment Overview

**Fig 1:**
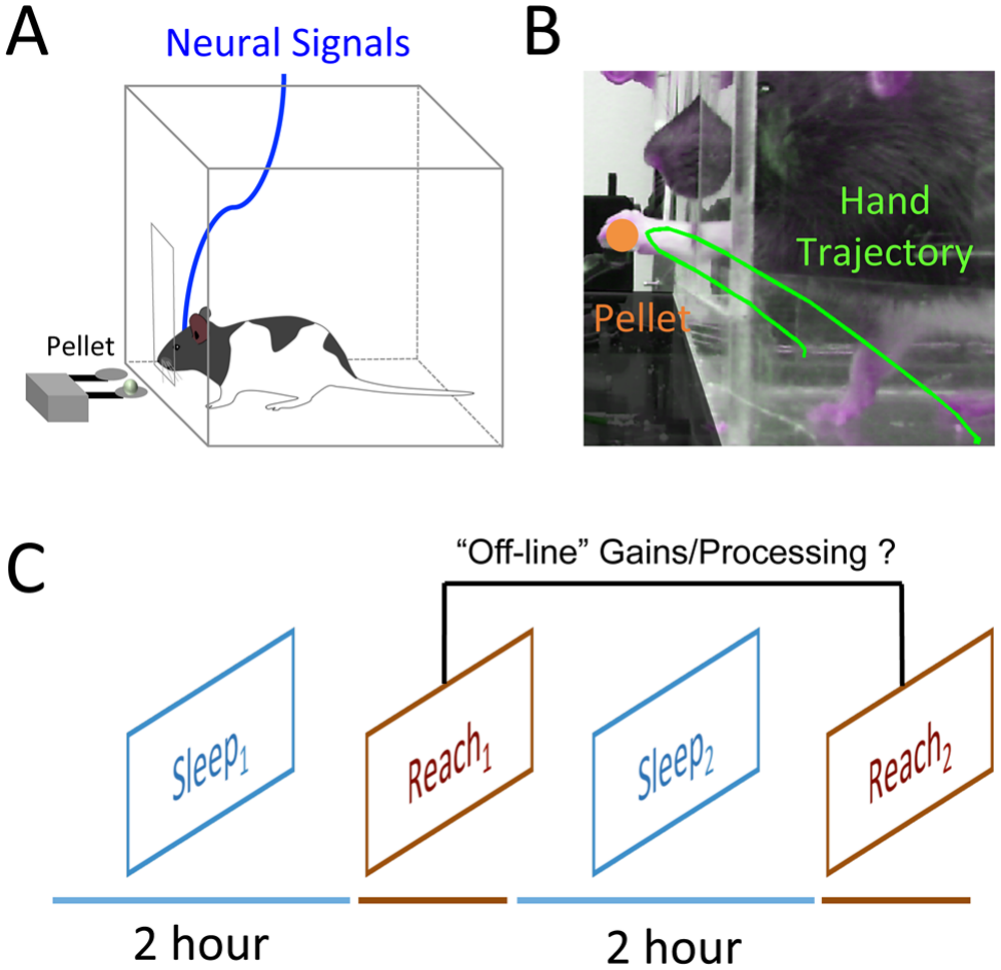
Behavioral paradigm. **(A)** Illustration of behavioral task [33], in which animals are required to reach through a narrow slit to grasp and retrieve a pellet into their cage. **(B)** Picture of animals grasping a pellet. Green line shows an example reach trajectory from which kinematic analyses are performed. **(C)** Overall behavioral/recording paradigm involved four blocks: a 2-h block of spontaneous recording (Sleep_1_), a block of skilled motor training (Reach_1_), another 2-h block of spontaneous recording (Sleep_2_), followed by a final reach block (Reach_2_).

Microelectrodes were implanted (tetrode and microwire arrays were used in different animals, see Materials and Methods) into the lateral part of the caudal forelimb area of rats, the region most strongly associated with fine motor control of the distal forelimb, and the region directly involved in plasticity following skilled motor learning [30–32] (S1 Fig). Five days after electrode placement, animals began skilled motor training (Fig 1A and 1B). Skilled motor learning was conducted using the Whishaw forelimb reach-to-grasp task [33,34]. We chose this task both due to homology to skilled learning tasks in humans [35,36] and the extensive evidence that this task is associated with multiple levels of neural plasticity, including changes in Long-Term Potentiation (LTP) [37], spine growth [38–40] and motor map plasticity [30,41,42]. Neural activity was monitored during the following sequence of blocks: a “baseline” sleep block (Sleep_1_), a skilled motor learning session (Reach_1_), a sleep block (Sleep_2_), and a subsequent learning block (Reach_2_) (Fig 1C). Thus, we were able to compare both how task-related neural activity was modulated after sleep (i.e., by comparing Reach_1_ and Reach_2_) as well as how motor learning affects neural activity during sleep (i.e., by comparing Sleep_1_ and Sleep_2_).

### Offline Gains in Skilled Motor Performance

**Fig 2:**
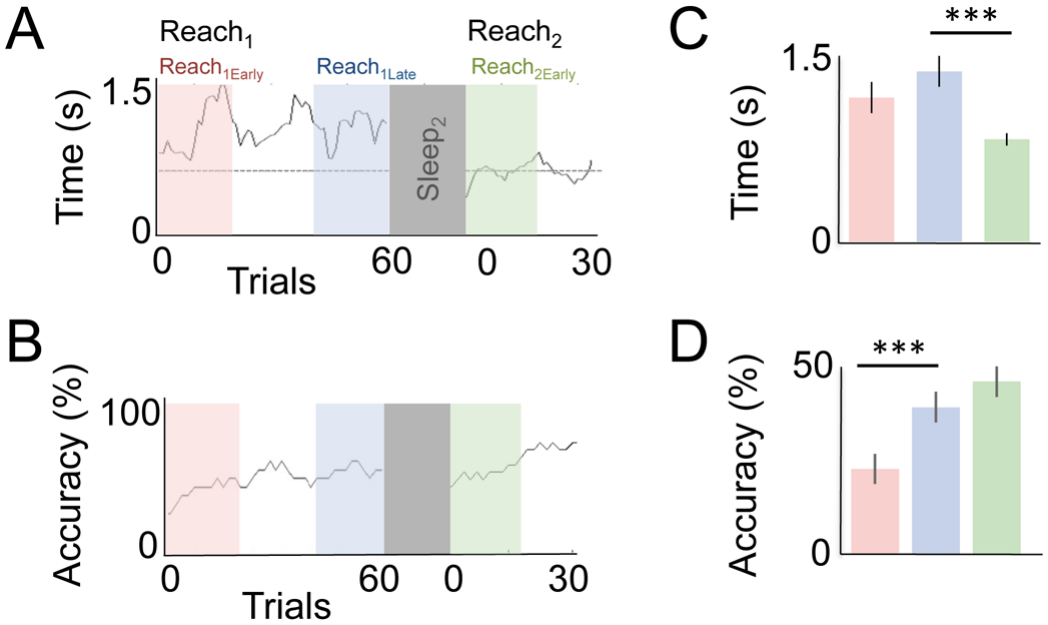
Comparison of online and offline changes in skilled motor behavior. Motor learning was characterized across two major parameters: speed and accuracy. **(A)** Speed was quantified for every trial by measuring the time from reach onset to retraction of forelimb. **(B)** Unlike changes in speed, animals showed a general improvement in accuracy across the learning block (Reach_1_), but accuracy was not significantly different after sleep (Reach_2_). For both plots we used a moving average window of ten trials. **(C,D)** Effects were quantified across all animals (*n* = 5). Error bars represent mean ± SEM; *** *p* < 0.001.

Motor skill learning is typically assessed across two dimensions: speed and accuracy [1,13,43,44]. We examined both here, measuring accuracy as percent success in retrieving the pellet and speed as the overall time the animal took to perform the full reach-grasp-retract motor sequence (Fig 2). Online changes in skilled motor performance were quantified by comparing the first 20 trials (hereafter “Reach_1early_”) to the last 20 trials (hereafter “Reach_1late_”) of the first learning session; offline gains were measured by comparing the last 20 trials from the initial learning session (i.e., “Reach_1late_”) with the first 20 trials from the subsequent reach block (hereafter “Reach_2early_”). Across all animals, we found significant online improvements in accuracy (Fig 2D, *p* < 0.001, Wilcox rank-sum test) in Reach_1early_ versus Reach_1late_; see Materials and Methods) but without improvements in speed (Fig 2C, overall ANOVA F(2,98) = 6.6, *p* < 0.01; post-hoc *t* tests comparing Reach_1early_ with Reach_1late_, *p* = 0.3) during the initial training session (Reach_1_). Following sleep, however, animals executed the entire movement sequence considerably faster (Fig 2C, post-hoc *t* test comparing between Reach_1Late_ and Reach_2early_, *p* < 0.001), with no decrements in accuracy (Fig 2D, *p* = 0.3, Wilcox rank-sum test between Reach_1Late_ and Reach_2early_). Thus, sleep appeared to increase movement efficiency and was associated with no decrement in accuracy.

To further probe the effects of sleep on motor performance, we also analyzed online and offline changes in the trajectory of forelimb movements. To perform this analysis, we calculated whether there was a change in movement trajectories either during online learning or after sleep (S2 Fig). Trajectories were calculated both using an “external frame of reference” (i.e., relative to the end-point position of the pellet) and an “internal frame of reference” (i.e., relative evolution of the trajectory after the start of movement). During online training, we found that animals changed their external frame trajectory, suggesting greater orienting towards the pellet at the start of the movement, without significantly altering the kinematics of the trajectory itself. In contrast, after sleep, while the externally referenced trajectory remained unchanged, the internally referenced trajectory kinematics appeared to be significantly changed. This suggests that different aspects of kinematics are modified during online versus offline processing.

### Offline Modulation of Task-Related Neural Activity after Sleep

**Fig 3:**
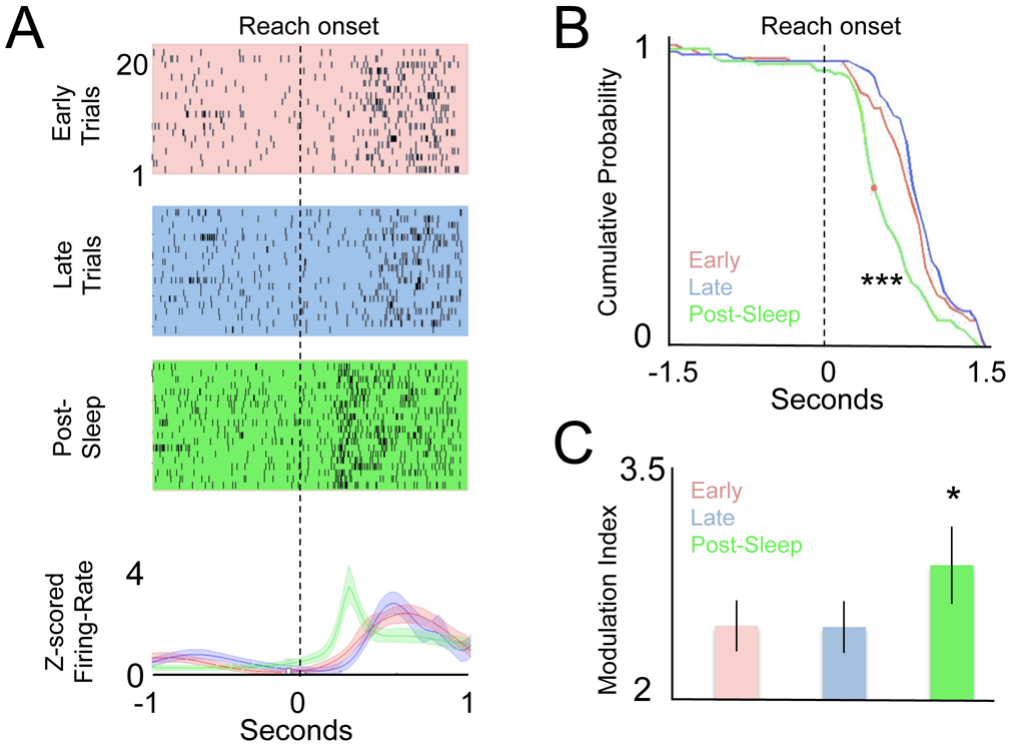
Comparison of online and offline changes in neural activation. **(A)** Change in movement-related activation of a single neuron during Reach_1early_, Reach_1late_, and Reach_2early_. Dotted line is the time of reach onset. Traces below represent a Bayesian adaptive regression spline fit of the respective PETH. **(B)** Curves show respective cumulative distributions of the single neuron PETH time to peak (red dot represents median timing). While there was not a significant change in the distribution for online learning (Kolmogorov-Smirnoff test, *p* = 0.9 comparing Reach_1early_ versus Reach_1late_, there was a significant shift after sleep (*p* < 0.001 comparing Reach_2early_ to both Reach_1early_ and Reach_1late,_ *n* = 4 animals, 99 units). **(C)** Change in task-related neural modulation. Task related modulation index is ratio of baseline firing to the peak instantaneous firing rate *n* = 4 animals, 99 units). Error bars show standard error of the mean (S.E.M.) * *p* < 0.05, *** *p* < 0.001.

To investigate how changes in neural activity underlie the offline gains in motor efficiency described above, we compared the task-related activity during Reach_1early_, Reach_1late_, and Reach_2early_. For each neuron, we calculated the peri-event time histogram (PETH, smoothed using a Poisson-based Bayesian-adaptive regression model [45]) time-locked to reach-onset (Fig 3A). Reach onset was defined by the start of physical movements (i.e., identical to those used to calculate the behavioral metrics above) and not based on external cues. During each of the early, late, and post-sleep blocks, we estimated the degree of modulation (i.e., the peak firing divided by the pre-reach baseline) and the time to peak firing. Even in the very earliest learning block (Reach_1_), 92/102 neurons showed some evidence of task modulation, defined as at least a 2-fold increase in firing rate compared to the baseline (S3 Fig). As with other studies that have examined neural recordings during forelimb reach tasks in rodents [46,47], we found that single units demonstrated time-locked activity across many phases of reach (S3 Fig; See figure legend for further discussion on the distribution of neural activity observed). Three units were excluded from further analyses because of very sparse firing, which made it difficult to properly estimate PETH and/or timing information.

We next analyzed online and offline changes in the task-related modulation of neural activity. Interestingly, we found a strong and specific effect of sleep in changing both the timing and magnitude of task-related activity at a single-unit level (example shown in Fig 3A). During online learning, the relative timing to reach-onset (Fig 3B, Kolmogorov-Smirnoff, *p* = 0.9) and task-related modulation (Fig 3C, overall repeated-measures ANOVA F(2,98) = 5.5, *p* < 0.01; *p* = 0.9, paired *t* test between Reach_1_ and Reach_2_) did not change significantly. However, after sleep, there was a strong and highly significant change in the time to peak of the PETH (i.e., “temporal coupling shift,” Fig 3B, Kolmogorov-Smirnoff *p* < 0.001) and a smaller but significant change in task-related modulation (Fig 3C, *p* < 0.05, paired *t* test comparing modulation for Reach_2early_ to both Reach_1_ blocks). Across all neurons, we also analyzed the mean shift following sleep. We found that there was a 200 ± 65 ms shift in the temporal coupling of neural activity to reach onset after sleep (repeated-measures ANOVA F(2,98) = 7.7, *p* < 0.001; *p* < 0.001 paired *t* test comparing modulation for Reach_2early_ to both Reach_1_ blocks). S4 Fig shows the entire distribution of shift in neural firing for neurons during both the online and offline periods, demonstrating a widespread effect across most neurons we recorded from.

### Sleep Is Essential for Offline Behavioral Gains and Neural Modulation

**Fig 4:**
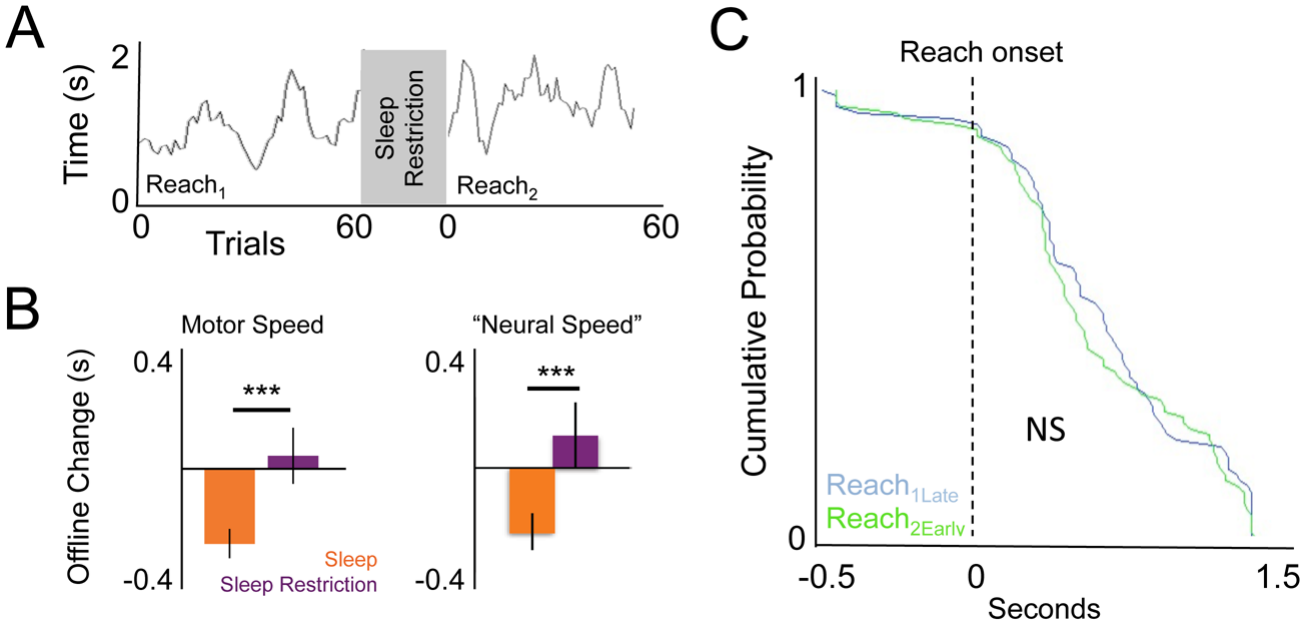
Sleep restriction prevents offline gains and temporal shifts. **(A)** Representative example of lack of improvement in speed after sleep-restriction (moving window average of 10 trials). **(B)** Comparison of effects of sleep restriction versus sleep (*n* = 5 each) on changes in motor speed and “neural speed” (i.e., shift in PETH peak; *n* = 99 neurons from sleep animals, *n* = 80 neurons from sleep-restricted animals). Error bars show S.E.M. *** *p* < 0.001 **(C)** Cumulative distribution of single neuron peak of PETH (non-significant based on Kolmogorov Smirnoff test).

Prior reports in humans have demonstrated that sleep is required for offline performance gains [9,13]. To study whether sleep is essential for the behavioral and neural changes described above in our experimental paradigm, we performed a follow-up experiment in which a new set of animals were given 2 h of sleep-restriction between Reach_1_ and Reach_2_ (see Materials and Methods). To assess how this modified offline changes in behavior and neural firing, we compared the last 20 trials in Reach_1_ with the first 20 trials in Reach_2_ (Fig 4A). We found significant differences in offline changes in movement speed (Fig 4B “motor speed,” *p* < 0.001, 2-sided *t* test comparing the sleep and sleep restriction groups). We also found significant differences in the offline changes in the temporal shifts of peak task-related activity when compared to animals allowed to sleep (Fig 4B “neural speed,” *p* < 0.01, 2-sided *t* test). Fig 4C also shows the cumulative distributions of the individual timings of neural PETHs both before and after the sleep restriction (Kolmogorov-Smirnoff *p* = 0.7 between Reach_1late_ and Reach_2early_). Thus, our data suggests that in the absence of sleep there were no changes in movement or neural speed, i.e., no offline gains at either a behavioral or neural level. It is also important to note that the sleep-restriction paradigm did not impair performance at either a behavioral or neural level; these animals were neither significantly slower (*p* = 0.7, one-sample *t* test comparing last 20 trials pre versus first 20 trials post sleep-restriction, *n* = 5 animals) nor significantly less accurate (*p* = 0.15, one-sample *t* test comparing accuracy in the last 20 trials before versus first 20 trials after sleep-restriction, *n* = 5 animals). Moreover, the population-tuning curve was not significantly different (Fig 4C). Thus, we did not observe any significant changes in performance (i.e., either a decrement or offline gains) after the period of sleep-restriction, but likewise, no offline gains in neural or motor speed.

### Extra Sleep Prior to Learning Does Not Promote Faster Movements

The experiments described above were conducted with animals that were allowed to sleep twice (i.e., Sleep_1_ and Sleep_2_), with offline behavioral changes occurring after the second sleep block. The sleep-restriction control described above indicates that such offline gains do not occur simply as a result of the passage of an equivalent period of time awake. However, it is possible that the effects observed (e.g., the changes in movement speed after Sleep_2_) occur simply as a result of the animals having more overall sleep before performing the task. In other words, because animals in our experimental group are able to sleep for two sessions, it is possible that what we interpret as offline gains are simply a product of normal practice-related improvements (i.e., during online training) that occur when animals are well-rested. To address this issue, we allowed more sleep prior to the initial training block. Thus, animals were allowed to undergo both Sleep_1_ and Sleep_2_ blocks before any training occurred (S5A Fig). Animals were awoken for 20 min between those two blocks and were given 50 pellets to generally mimic the task structure of the first set of experiments. Animals were then given 150 trials of reach training to assess whether having extended sleep prior to training would result in improvements in motor speed during the training sessions itself. We found that extra sleep prior to training was not sufficient to induce significant changes in speed during training (S5B–S5D Fig, *p* = 0.36, paired *t* test, comparing the first 20 and last 20 trials, *n* = 5 animals). Animals were also tested the next day (i.e., after 24 h), to observe whether they showed similar offline gains as described in our early experiments. Indeed, these animals showed offline improvements in speed (*p* < 0.0001, paired *t* test comparing last 20 trials from Day 1 and the first 20 trials on Day 2, *n* = 5 animals) with maintenance of accuracy (*p* = 0.1, paired *t* test comparing last 20 trials from Day 1 and the first 20 trials on Day 2, *n* = 5 animals). This control experiment further confirms the role of offline processes in mediating speed improvements during skill acquisition.

### Reactivation of Neural Ensembles during Sleep Drives Subsequent Modulation of Task-Related Neural Activity

Having demonstrated large-scale, sleep-dependent changes in neural activity and related motor behavior, we next examined what neural processes may be mediating these effects during the sleep block. We hypothesized that replay of task-related neural ensembles during NREM sleep drives the offline behavioral gains and neural modulation previously described. To investigate this, we used principle components analysis to identify task-related patterns of neural activity (i.e., neural ensembles) and then probed replay of these ensembles during sleep using methods described previously [29,48,49]. Across animals, mean time spent in NREM sleep during the Sleep_1_ block was 28.6 ± 11.3 min, and the mean time spent in NREM sleep during Sleep_2_ block was 24 ± 4.1 min (paired *t* test = 0.6).

**Fig 5:**
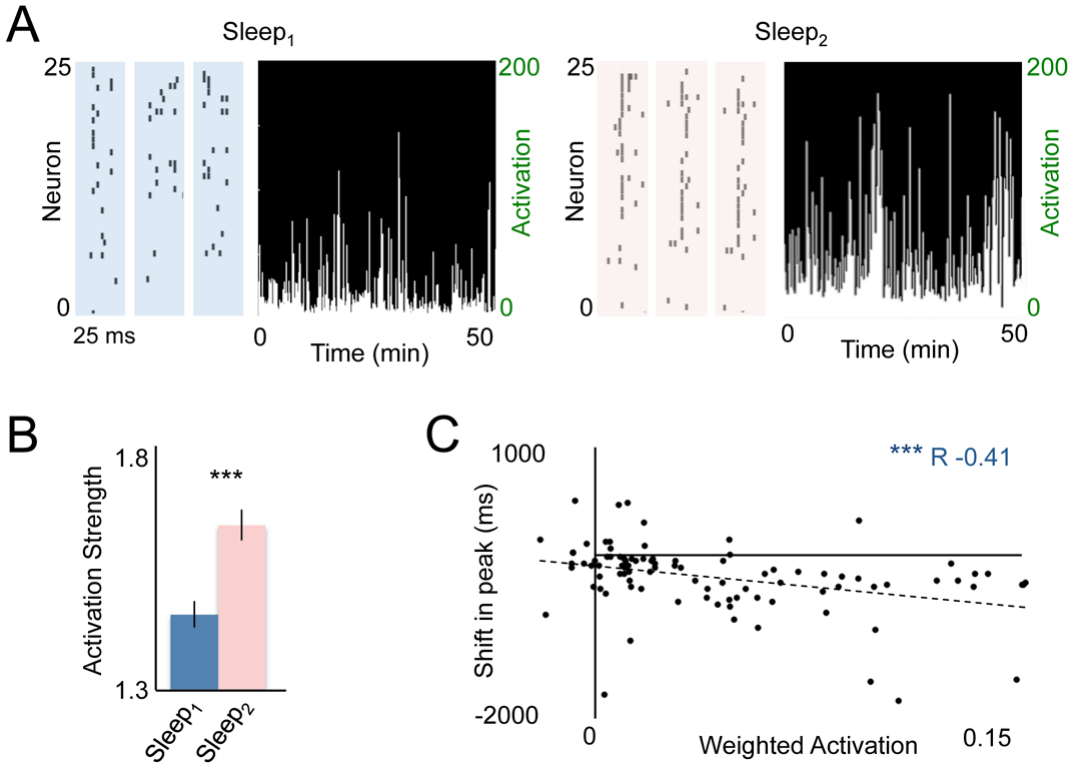
Reactivation of task-related neural ensembles during slow-wave sleep. **(A)** Example of reactivation events prior to and after learning (i.e., Sleep_1_ versus Sleep_2_ events in respective blue and red boxes). Also shown are the activation strengths of reactivation events during the initial NREM epochs from Sleep_1_ and Sleep_2_. **(B)** Across all animals, there was a significant reactivation of task-related ensembles (*p* < 0.001, sign-rank test). Quantification was based on the entire recorded NREM sleep. **(C)** Linear correlation between single neuron reactivations and neural modulation during Reach_2_. To estimate “single neuron reactivation,” we first calculated the overall ensemble reactivation [(mean activation during NREM from Sleep_2_)–(mean activation during NREM from Sleep_1_)] and then multiplied this value with the principle component weight for each neuron. Plot shows the regression analysis between each neuron’s reactivation and subsequent temporal shift (r = -0.41, *p* < 0.001). Error bars show S.E.M. * *p* < 0.05, ** *p* < 0.01,*** *p* < 0.001.

Ensemble reactivation during sleep was quantified by applying a template created using principle components analysis (PCA) of the task-related neural activity. In other words, PCA was used to create templates that captured patterns of synchronized neural activity during task performance (S6 Fig). PCA resulted in a number of principle components (hereafter termed “ensembles”) that reflected patterns of common variance across the recorded single-units, with each component comprised of weights that reflected the contribution of each neuron to that particular ensemble. To represent the activity of a particular ensemble the traditional method is to multiply the weights from each neuron in a particular ensemble with the Z-scored activity matrix [48]. This same method can be used during sleep to assess the degree to which this ensemble is being reactivated (S6 Fig) [29,50]. Specifically, the ensemble defined from the task was multiplied by the Z-scored neural activity recorded during sleep blocks, resulting in a one-dimensional vector that represented the “activity” of that ensemble during the sleep blocks before and after (Fig 5A). Reactivation was thus defined as increased “activity” of the ensemble during the sleep-block after learning compared to the sleep-block prior to learning (Fig 5A–5C). In this relatively rapid motor task (~1 s), the first ensemble captured more variance than any other component and we therefore focused our analysis on it. Prior reports using this method have found that weaker ensembles (i.e., those with lower eigenvalues) show limited evidence of reactivation [50]. When we did examine the second ensemble, we found considerably more variability in terms of reactivation across animals (i.e., only some animals showed evidence of reactivation of this ensemble).

After learning, activation strength was significantly stronger during NREM sleep blocks for these task-related ensembles in comparison to the sleep block that occurred before learning (Fig 5B, *p* < 0.001, **Wilcox** non-parametric sign-rank test). We originally hypothesized a significant relationship would occur between reactivation of neural ensembles and subsequent temporal shifts of cortical neurons. To evaluate whether such a relationship existed, we calculated the ensemble reactivation for each unit. Reactivation was defined by calculating the difference in activation strength between Sleep_1_ and Sleep_2_ for that ensemble (i.e., Sleep_1Activation−_Sleep_2Activation_), multiplied by the principle component (PC) score for each neuron in that ensemble, resulting in that neuron’s “weighted” reactivation score. Interestingly, we found that the degree of reactivation at a single neuron level during sleep strongly and significantly predicted the increase in temporal coupling to reach onset that occurred upon awakening (Fig 5C, r = -0.41, pearson correlation, *p* < 0.001, analysis conducted across all neurons, *n* = 4 animals). This indicates that neurons that experienced the strongest reactivation during the NREM sleep block also experienced the greatest shift in temporal coupling to reach onset during the skilled reach task upon awakening.

### Replay of Motor Sequences during Reactivation Events

To better understand the relationship between reactivation events and task-related neural activity, we performed two additional analyses (S7 Fig). First we performed an analysis to demonstrate that the reactivations truly reflect a specific pattern of task-activity—in other words, that the same neurons active during the task are highly active during the replay event. To perform this analysis, we calculated PETHs for each neuron during the ensemble replay (**binned at 25 ms**, and including data 250 ms before and 250 ms after each replay event, using only the top 10% of activation strengths) and compared this with the PETHs and PC ensemble created from activity during the reach task (S7A and S7B Fig). Importantly, this binning allows us to estimate variability in firing rates across neurons during reactivation events, but not temporal variability across neurons. Each PETH was then sorted according to the PC weight extracted during the task block. As predicted, there was good correspondence between the PC weight and the degree to which these neurons were firing during the reactivation (S7C Fig). This provides evidence that reactivations represent synchronous co-activation specifically of task-related neurons; in other words, variation in firing rates during the task are observed as variations in synchronous firing during reactivation events during sleep.

Prior studies have distinguished “reactivation,” observed as synchronous activity of task-related ensembles during subsequent sleep periods [50], and “replay” which involves a recurrence of sequential activity during subsequent sleep epochs. To assess the degree to which there is replay, we next examined the microstructure of reactivation events at a single millisecond resolution. To perform this analysis, we used a previously described template matching technique [51]. To create the task-template, we extracted the PETH both 250 ms prior to and 1,000 ms after the reach onset in each animal. This activity pattern was binned at different resolutions (50 ms, 125 ms, 250 ms, and 1,250 ms), to create bin templates ranging in size from 25 bins down to 1 bin (S7D Fig). Importantly, each binning resolution contained less temporal structure (i.e., the 1,250 ms bin does not retain any temporal information). The template matrix (neurons x bin) was then correlated with single-unit activity patterns that occurred during reactivation events identified above. Reactivation events were kept at a 1 ms resolution (i.e., no binning) for the template matching procedure. We found a general increase in the degree of template correlation after learning (*p* < 0.0001 across every template studied, S7D Fig). However, the highest degree of correlation and the greatest change post occurred with the largest bin size (i.e., with the least temporal information). This suggests that even while there is a small but significant change in the temporal structure of reactivation events, they are more linked to synchronous activation of neurons that fired during task performance (another example shown in S7E Fig).

### Relationship of Reactivation Events to Spindles/Slow-Wave Oscillations

**Fig 6:**
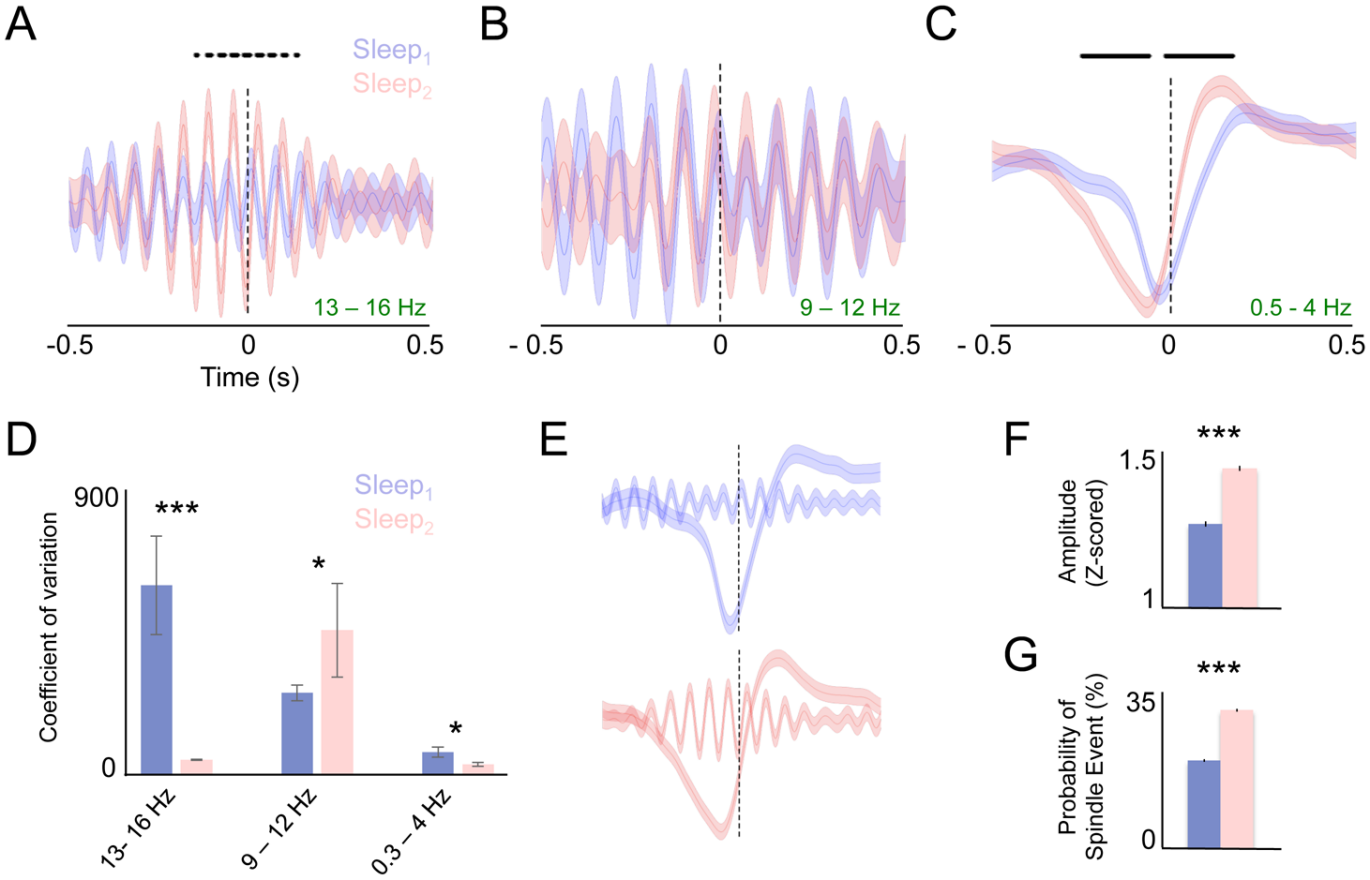
Relationship of ensemble reactivation to NREM oscillations. **(A)** Event-triggered z-scored LFP for fast-spindle oscillatory frequencies filtered at 13–16 Hz. Post-hoc differences (*p* < 0.05) indicated by points above graph. **(B)** Event-triggered LFP for slow-spindle frequencies (9–12 Hz). There were no significant differences across any time point. (**C)** Event-triggered LFP for slow oscillation frequencies (0.5–4 Hz). **(D)** Comparison of the coefficient of variation (CV) for each oscillatory band. For fast spindles and slow oscillations, for each point that was significantly different between the two groups, we compared the CV. After learning, the CV was significantly reduced, suggesting more consistent temporal locking after learning. For slow-spindle oscillations, because no points were different between the two groups, we instead used the points that were significantly different in the fast-spindle frequency. **(E)** Comparison of the z-scored temporal coupling between reactivation-triggered slow wave and fast-spindle oscillation before and after learning. **(F)** Changes in mean event-triggered spindle amplitude. We calculated the instantaneous analytic amplitude of the fast spindles at the slow-wave trough for each reactivation. There was a significant increase in spindle amplitude. **(G)** Increase in the probability that a reactivation event was associated with a fast-spindle oscillation. Error bars show S.E.M. * *p* < 0.05, ** *p* < 0.01,*** *p* < 0.001.

Prior studies have suggested that both spindles and slow-wave oscillations may mediate offline gains in motor performance [9–11,13]. To further probe the relationship between ensemble reactivations and these phenomena, we calculated the event related local field potential (LFP) locked to the top tenth percentile of reactivation events after sleep (Fig 6). We first calculated the event-triggered average (ETA) of LFP filtered at slow/delta-oscillations (i.e., 0.5–4 Hz). We next subdivided spindles into slow and fast frequencies because of prior evidence suggesting that fast-spindles in particular are specific to offline gains [10,51]. We thus examined the ETA of slow-spindles (filtered at 9–12 Hz) and fast spindles (filtered at 13–16 Hz). Interestingly, we found an increase in the locking of fast but not slow spindles with reactivation events after learning (Fig 6A and 6B, * above indicates significant post-hoc differences using a paired *t* test). Likewise, we saw a change in the association of these events with slow oscillations (Fig 6C). We probed phase-locking in two different ways. First, we calculated the coefficient of variance across events for those time points that were significantly different in Sleep_1_ versus Sleep_2_. As expected, we found a significant reduction in the coefficient of variation (CV) across these time points for fast-spindles and slow-oscillations (Fig 6D, *p* < 0.0001, ranksum test). Because there were no significant differences in the slow-spindle frequencies, to assess CV we used the same time points as used for fast-frequencies. We found a slight increase in the CV (Fig 6D, *p* < 0.05 ranksum test), suggesting that the increased locking is specific to fast-spindle oscillations and not a general phenomenon. We also calculated the instantaneous phase at the slow and fast spindle oscillation, and the slow-wave oscillations at t = 0 (i.e., when the reactivation event occurred), using circular statistics comparisons [52]. Prior to learning, there was no significant phase-relationship between fast-spindles and reactivation events. However, after learning, the mean phase (in radians) of the fast-spindle oscillation at the time of these reactivation events -2.06, (95% confidence interval [CI] of .5542). There was not a significant phase-relationship between reactivation events and slow spindle oscillations. Finally, there was evidence for a slight but significant phase-shift after learning in coupling with slow-oscillations. Prior to learning, the mean phase of the slow oscillation at the time of reactivation was -2.395 (95% CI .0953, using circstats toolbox); after learning, the mean phase at the time of reactivation was -2.07 (95% CI .077, using circstats toolbox), a highly significant difference (*p* < 0.0001 ANOVA using circstats toolbox). These analyses demonstrate that, independent of changes in the mean amplitude, after learning there was significant phase-coupling to the spindle-oscillations, and a significant phase shift relative to the slow-wave oscillation, and these changes partly explain the results observed in Fig 6A–6D.

These results suggest that following learning, high reactivation events are more strongly coupled to both fast spindle oscillations and slow oscillations (Fig 6E). To probe this further, we calculated the instantaneous analytic amplitude of the local field potential filtered in the fast-spindle frequency at the trough of the reactivation-triggered slow-oscillation for reactivation event. Across events, we found a strong and significant increase in the analytic amplitude at this time-point (Fig 6F, ***p* < 0.001, Wilcox** ranksum). Finally, using an automated detection algorithm, we assessed whether there was a significant change in the proportion of distinct spindle events associated with these high strength reactivation events. Specifically, we analyzed the LFP filtered in the fast-spindle frequency time-locked to high-reactivation events pre/post learning, to assess whether there was an increase in the proportion of spindles associated with the highest reactivation events after learning. We found that after motor learning, spindles were 60% more likely to occur in association with these high reactivation events compared to before learning. (Fig 6G, ***p* < 0.001**, Wilcox ranksum).

### Lack of Reactivation during Later Stages of Learning

**Fig 7:**
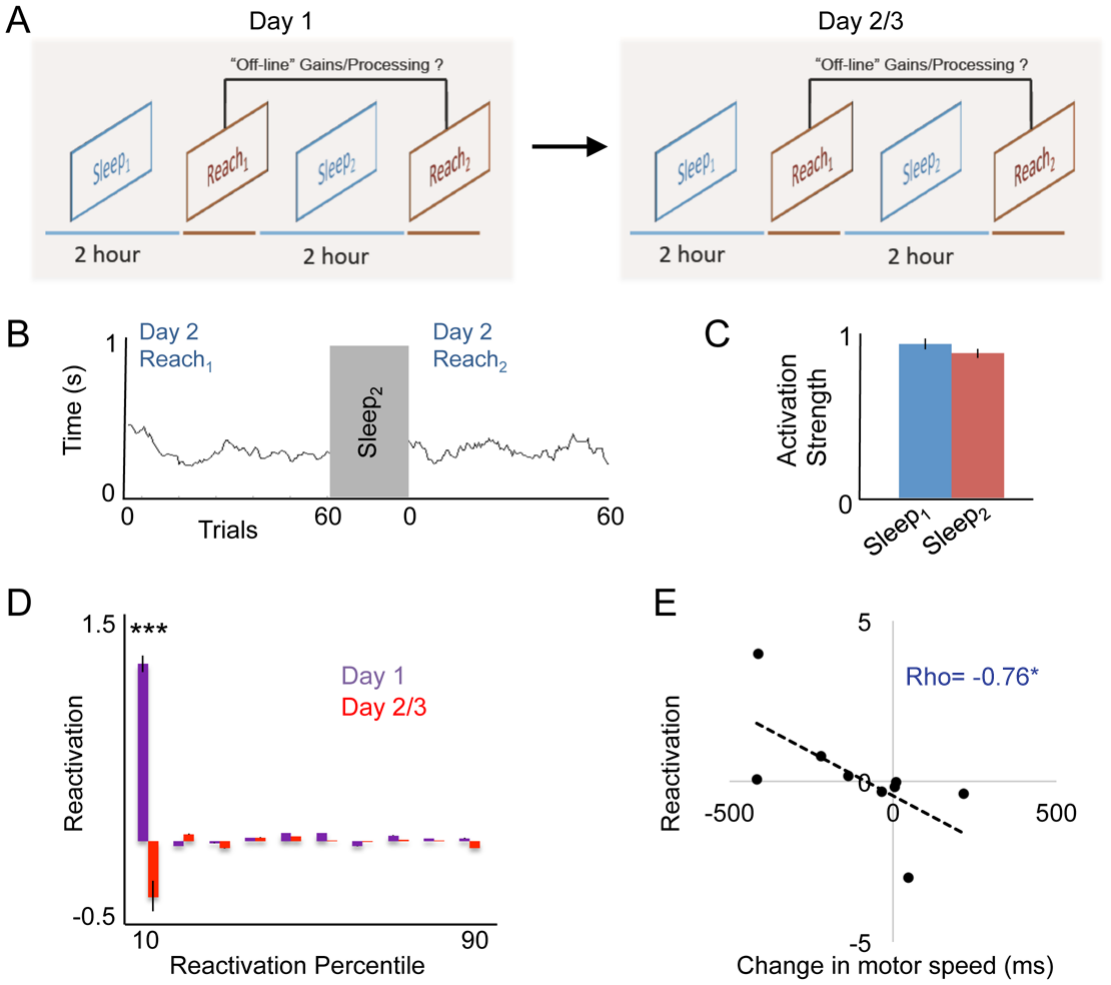
Lack of increased reactivation with continued motor learning. **(A)** We performed the same experiment as described for day 1 (Sleep_1_/Reach_1_/Sleep_2_/Reach_2_) on subsequent days to assess whether there was evidence of continued reactivation on these days. **(B)** Movement speed trends during Reach_1_ and Reach_2_ on day 2 for one animal. **(C)** Comparison of respective reactivations for subsequent training days (sign-rank *p* = 0.4). **(D)** Comparison of reactivations on day 1 versus subsequent days grouped into deciles. Mean differences were compared for each decile (*** *p* < 0.001). We found significant differences between groups at every decile, though clearly the greatest difference occurs at the “top” decile of reactivation events. **(E)** Correlation between top ten percentile reactivation events and offline changes in movement speed. We found a significant correlation (Spearman’s Rho = -0.76, *p* < 0.05) in which reactivation predicted offline motor improvements. Error bars show S.E.M. * *p* < 0.05, ** *p* < 0.01,*** *p* < 0.001.

We next analyzed patterns of activity during “later-stages” of learning, specifically defined here as continued practice on the skilled motor task on subsequent days. Prior research has divided motor learning into an early phase associated with the establishment of gross kinematic patterns and rapid gains in motor performance, and a later phase associated with overall kinematic stability and slower incremental gains in skill acquisition [5]. We have so far demonstrated that reactivation of task-related ensembles occurred after the initial motor learning session, when animals appeared to first form and then consolidate a novel kinematic trajectory. To explore whether changes in reactivation continues to occur through later stages of motor practice, we investigated whether task-related neural activity continued to experience an increase in the reactivation strength on subsequent days of motor learning (Fig 7A). For this analysis, data was gathered from three animals across two additional days of motor training. As there were no significant differences between day 2 and 3 on any of the parameters assessed below, data was pooled across these two days for the purposes of this analysis.

On subsequent sessions of motor learning (i.e., conducted on days 2–3), there were no further offline changes in movement speed/efficiency (example of one animal Fig 7B, *p* > 0.8 comparing Reach_1Late_ and Reach_2Early_ across all animals), indicating overall stabilization of the kinematic pattern. Despite this, there was continued evidence of learning, i.e., accuracy improved by 33% during the reach training in these subsequent sessions *p* < 0.05 Wilcox rank-sum test. The stable kinematics and slow improvements in accuracy are largely consistent with these subsequent training days being part of the late/slow-period of motor learning.

During this later phase of learning, we found no evidence of increased reactivation strength of task-related ensembles during sleep (Fig 7C, sign-rank *p* = 0.2). To assess whether this lack of an effect was significantly different compared to changes observed on day 1 of motor learning, we next calculated the overall reactivation (rank-ordered Sleep_2_–Sleep_1_ activations for each animal) on day 1 and compared this with reactivation on subsequent days of training. In addition, because prior analyses [29,50] have suggested that there is a highly skewed distribution of ensemble activations during sleep (i.e., many low-value activations), we further subdivided reactivation differences according to the overall percentile strength and compared across deciles (Fig 7D). This analysis demonstrates the highly skewed nature of these re-activations while also demonstrating the lack of an effect in later motor learning periods. Finally, across behavioral sessions in these animals over the first 3 d, we found a significant correlation between the degree of reactivation observed (using the top 10% reactivations pre versus post learning) versus changes in the motor speed before and after sleep (Spearman correlation, Rho = -0.76, *p* < 0.05, Fig 7E).

## Discussion

Many previous studies have examined the role of sleep in promoting offline gains in motor skill performance, with NREM sleep in particular promoting various types of motor learning [9,13,14,16,17,53,54]. However, little was known about the neurophysiological processes by which NREM sleep mediated motor memory consolidation. Here we show that reactivation of task-related neural patterns during NREM sleep is explicitly related to both performance improvements and plasticity of neural responses. We specifically found that these high reactivation events were closely linked to an increase in fast spindle oscillations and became slightly phase-shifted relative to the slow oscillations. Finally, we found that neural reactivation is very specific to early motor learning, and not simply a reflection of motor practice; NREM-sleep reactivation was not evident during later stages of motor learning once kinematic patterns had been stabilized. These results suggest that task-related neural reactivation during NREM sleep plays a key role in stabilizing the basic motor pattern during motor learning, with subsequent improvements not dependent on large-scale reactivation during sleep.

We show here that sleep-dependent reactivation of neural ensembles occurs in the context of procedural learning. Prior demonstrations of reactivation in cortex have been described in visual [21] and prefrontal [20,50,55,56] cortex. Importantly, these studies occurred in the context of hippocampal-dependent behavior and primarily in coordination with replay events from the hippocampus [20,21,50,55,56]. Another demonstration of sleep-dependent reactivation, by our group, occurred in the context of neuroprosthetic learning [29]. While neuroprosthetic learning is beginning to be explored in more detail [57–59], it is unclear whether this learning represents declarative, procedural, or some more abstract form of learning that is fundamentally different than either of the above. We thus identify the role of neural reactivation measured at single-neuron resolution for motor memory consolidation.

Prior research has divided motor learning into an early phase, associated with the establishment of gross kinematic patterns and rapid gains in motor performance, and a later phase, associated more strongly with subtle refinements of kinematic patterns and more incremental changes in skill acquisition [1,2,5]. We show here that large-scale reactivation of neural ensembles is associated with kinematic improvements and related changes in neural activity patterns. Indeed, our study suggests that offline processing during sleep may play a key role in the consolidation of motor memories, thus allowing animals to transition to a later phase of learning that is more strongly associated with more subtle motor refinements and increased automation [60].

We also found that after sleep there was greater temporal binding of single-unit activity in motor cortex to the initiation of the reach movement. We hypothesize that the faster activation of neurons after sleep are related to the binding of separate motor programs encoding specific parts of the complex movement (i.e., “reach, grasp, retract”), into one integrated motor program. While our evidence suggests that motor cortex is the final output of this program, it may not be where this program is ultimately “stored;” in other words, the ensembles controlling the different motor actions may be bound together by intrinsic connectivity within motor cortex or may be activated through distributed circuits that occur across cortico-striatal or prefrontal circuits [61]. It is important to point out that in hippocampal-dependent replay, systems consolidation theory would suggest that memories are being transferred from subcortical to cortical representations. In motor learning, it may well be that memories are being transferred from cortical to subcortical representations, perhaps representing a fundamentally different role for reactivation. Further research will be required to assess these ideas.

Studies conducted in both rodents and humans have demonstrated that early motor learning and motor cortical activity itself involves a distributed set of circuits including attentional/prefrontal regions, as well as cerebellum and dorso-medial striatum (i.e., the associative, prefrontal-connected portions) [3,12,60,62–68]. By contrast, later phases of skill learning seem to be more strongly associated with changes within motor cortex [31,41,64,64] and between dorso-lateral striatum (i.e., the “motor” striatial circuit) [62,67–68]. This suggests that ensemble reactivation during sleep, which seems to occur most strongly in association with this early learning phase, may occur through distributed reactivation across cortical and subcortical regions involved in various aspects of these motor actions.

This theory that motor ensembles are being driven by large-scale distributed networks during early motor learning processes, with stabilization associated with transfer of procedural memory into motor cortex, is consistent with what is known about how hippocampal associative circuits drive cortical replay in NREM sleep following declarative memory paradigms [22–24]. Moreover, this theory is also consistent with a recently proposed “active systems consolidation theory” [26,28] that suggests that spindle-dense Stage-2 sleep [28] serves to synchronize global cortical processes, thus mediating long-range consolidation of synaptic plasticity [18,19] and memory transfer to distant cortical regions [26]. Further studies involving dual recordings from disparate cortical regions during early/late motor learning sessions and during sleep will be required to demonstrate that ensemble reactivation truly is associated with active consolidation across a distributed set of cortical/subcortical brain regions.

Interestingly, we found a specific involvement of fast spindles occurring during slow-wave oscillations in the reactivation of task-related neural ensembles. While largely consistent with a body of work demonstrating the involvement of fast spindles in motor learning [10,11,51], this was quite distinct from what had been observed across the two previous studies demonstrating cortical reactivation in rodents [29,50]. In those studies, cortical reactivations seemed to be coupled most strongly to the peak negativity of the slow-oscillation [29,50] and in the case of prefrontal cortex, also to hippocampal sharp-wave ripples [50], which themselves occurred near the peak negativity of slow-wave oscillations and prior to spindle oscillations. By contrast, here we find that after motor learning, reactivation events seemed to be particularly time-locked to fast spindles that occur a short time after the trough of slow delta waves. This difference may represent an important and fundamental difference between motor and other forms of learning. Indeed, one recent study has demonstrated that during the trough of slow-wave oscillations, cortical neurons are driven strongly by hippocampal circuits whereas during spindle events hippocampal circuits are suppressed and processing is driven strongly by thalamo-cortical circuits [69]. Given that the bulk of non-cortical motor processing (i.e., from the cerebellum and striatum) are transmitted back to the cortex through the ventral thalamus [69], it is certainly plausible that emergent task-related ensembles during NREM sleep may be locked to spindle events, particularly if this reactivation is being driven by distributed mechanisms as previously postulated.

Together, our results shed light on the neural processes associated with offline gains in skilled motor performance. We have identified a specific neural correlate of the widely observed sleep-dependent improvement in movement efficiency and linked them to sleep dependent reactivation of activity patterns established during online earning. Our results particularly emphasize the importance of sleep during early motor learning when motor sequences are initially established. This phenomenon might be most relevant to early skill building during musical or sports training, or during early child development, when essentially all skills across both sensory and motor domains are new [54,70]. In addition, these results suggest a potential mechanism by which NREM sleep may serve to enhance plasticity and functional recovery following brain injury [71].

## Materials and Methods

### Animals/Surgery

This study was performed in strict accordance with guidelines from the USDA Animal Welfare Act Regulations and United States Public Health Science (PHS) Policy. The protocol was approved by the San Francisco VA Medical Center Institutional Animal Care and Use Committee (IACUC, Protocol Number 13–006). We used 15 adult Long–Evans male rats (approximately 8 wk old; see S1 Table for complete details of animals, units, etc.). Animals were kept under controlled temperature and a 12–h light, 12–h dark cycle with lights on at 06:00 A.M. Probes were implanted during a recovery surgery performed under isofluorane (1%–3%) anesthesia. The post–operative recovery regimen included administration of buprenorphine at 0.02 mg/kg b.w. and meloxicam at 0.2 mg/kg b.w. Dexamethasone at 0.5 mg/kg b.w. and Trimethoprim sulfadiazine at 15 mg/kg b.w. were also administered post–operatively for 5 d. All animals were allowed to recover for 5 d prior to start of experiments.

### Electrophysiology

We recorded extracellular neural activity using both tungsten microwire electrode arrays (MEAs, *n* = 3 rats, Tucker–Davis Technologies or TDT, FL) and tetrodes (*n* = 4 rats, Neuronexus, Michigan). Arrays were implanted in the caudal forelimb area of primary motor cortex (M1), centered at 3–4 mm lateral to bregma, 0.5 mm anterior to bregma to target upper limb primary motor cortex (M1) (S1 Fig). Final localization of depth (1,000–1,500 μm) was based on quality of recordings across the array at the time of implantation. We recorded spike and LFP activity using a 128–channel TDT–RZ2 system (Tucker–Davies Technologies). Spike data was sampled at 24,414 Hz and LFP data at 1,018 Hz. ZIF–clip based analog headstages with a unity gain and high impedance (~1 GΩ) were used. Only clearly identifiable units with good waveforms and high signal-to-noise were used. MEA recordings were sorted offline using PCA-based algorithms followed by manual cluster-cutting using TDT’s OpenSorter software. Tetrodes were sorted using “UltraMegaSort” toolbox (available online at https://physics.ucsd.edu/neurophysics/software.php), a set of MATLAB based scripts for tetrode sorting described in detail previously [29,72]. Specifically, a voltage-based threshold was set based on visual inspection for each channel that allowed for best separation between putative spikes and noise (typically this threshold was 4.5–5 standard deviation [SD] away from the mean). Snippets of data that crossed threshold were time-stamped as events, and waveforms for each event were peak aligned. K-means clustering was then performed across the entire data matrix of waveforms (30 samples/ch x 4 chs x # of waveforms). Automated sorting was performed by: (1) first over clustering waveforms using a K-means algorithm (i.e., split into many mini-clusters), (2) followed by a calculation of interface energy (a nonlinear similarity metric that allows for an automated decision of whether mini-clusters are actually part of the same cluster), and (3) followed by aggregation of similar clusters. Such aggregation allows for a reduction in the total numbers of clusters that need to be manually inspected. Automated sorting was followed by manual inspection and sorting of spikes (including further merging or splitting of automatically identified clusters and removing significant outliers based on Gaussian distribution of PC space), using feature space, auto-correlations, cross-correlations and linear discriminant analysis to determine which clusters represent single units and to prevent over-sorting (S8 Fig). Trial-related timestamps (i.e., trial onset, trial completion and timing of when animals reach the pellet) were sent to the RZ2 analog input channel using an Arduino digital board and synchronized to neural data.

### Behavior

Prior to surgery, animals were handled and acclimated to behavioral boxes and oriented to the pellet tray for 1 wk, at the end of which they were evaluated on 10 trials of the Whishaw forelimb reach to grasp single pellet task to determine handedness. This was followed by electrode implantation on the contralateral motor cortical hemisphere as described above. Five days after electrode implantation, animals were food-restricted for 2 d, followed by feeding animals a fixed amount during the course of training (2 average sized food pellets/day). Whishaw forelimb-reach was conducted using a clear plexiglass chamber, with a 1.5 cm slit for animals to place their forelimb through in order to reach a 45 gm pellet on a shallow dish 1.5 cm away from the front of behavioral chamber, using an automated chamber described in more detail in [34] (Fig 1A and 1B). Animals typically performed from 100–150 reaches in the first reach block (Reach_1_) and 50–75 in the re-test block (Reach_2_). All reaches were videotaped for post-hoc analysis of accuracy, kinematics, and dynamics.

All behavioral sessions began in the morning and consisted of 2 h of spontaneous recording (to record a “baseline” sleep period, Sleep_1_); motor skill learning (Reach_1_); a second 2-h block of spontaneous recording (Sleep_2_); and finally a “re-test” motor skill block to assess for changes in behavior/neural activity after sleep (Reach_2_).

### Sleep Restriction Paradigm

Sleep-restriction experiments were conducted similarly to the experiments described above, with the exception that during the second 2-h block of spontaneous recording (termed Sleep_2_ above), animals were kept awake. Specifically, sleep-restriction sessions began in the morning and consisted of 2 h of spontaneous recording (to record a “baseline” sleep period, Sleep_1_), motor skill learning (Reach_1_), a second 2-h block of sleep-restriction (Sleep Restriction), and immediately after this a re-test motor skill block to assess for changes in behavior/neural activity after sleep (Reach_2_).

For the sleep restriction experiments, animals were kept in the behavioral box in which they conducted initial training sessions. The animals were closely observed for any behavioral evidence of sleep. In addition, the LFP was monitored in real-time to detect any evidence of sleep signatures. If detected, we gently tapped the box to keep awake. Tapping was typically required less than 1x/min early during the restriction period; and by the end of the restriction period had escalated to around 3 taps/min to keep animals awake. The entire paradigm was carried out identical to the training paradigm. Specifically, neural data was recorded during a 2-h block of spontaneous activity, during which time animals were allowed to sleep. Subsequently, animals performed the skilled motor learning task. After this, we performed sleep restriction for a 2-h period, after which animals were re-tested.

### Analysis

Data analysis was performed using a combination of custom written scripts in MATLAB and toolboxes developed for neural analysis.

### Behavioral Analysis

We compared changes in task performance between and across sessions. Specifically, we compared the performance change between early and late trials by comparing changes in behavior between the first 20 and last 20 trials in block 1, and the effects of sleep by comparing the last 20 trials in block 1 with the first 20 trials in block 2. Three different aspects of learning were measured: accuracy (here defined as successful retrieval of the pellet into the chamber), speed (defined as time from the beginning of the reach to the pellet, and through to the execution of retract movement), and finally similarity of movements, assessed by calculating the Pearson correlations between movement trajectories in X-Y space. For this analysis, within each block (i.e., Reach_1early_, Reach_1late_, and Reach_2early_, we correlated movements in both the X-direction and Y-directions and averaged this together to get a trial × trial correlation matrix. We then calculated the mean correlation across all trials within the different blocks. Across all animals, statistical changes in accuracy were assessed by assigning, for each trial, a “1” for correct trials in which animals successfully retrieved the pellet and a “0” for incorrect trials, followed by logistic regression analysis of the overall distribution of 1’s and 0’s across groups. Changes in speed (measured as changes in time for the overall reach trajectory) were assessed using ANOVA/post-hoc Fisher’s test; and changes in Pearson correlations were calculated using ANOVA/post-hoc Fisher’s test after first Z-transforming correlation coefficients.

### Trajectory Analysis

In addition to the above analyses, we also conducted trajectory analyses in “state-space” using two different procedures. First, trajectories were performed without any processing, termed here an “external frame of reference.” For this analysis, we analyzed the distribution of Y-coordinates relative to X-coordinates, as a way of determining overall differences in the state-space of the trajectory. Statistical analysis of these distributions across the different groups of trials for each X-coordinate (binned into units of 4; Reach_1Early_ Reach_1Late_ and Reach_2_) was performed using bootstrap techniques (confidence intervals were estimated by performing random sampling with replacement 2,000 times for each X-coordinate being estimated). We also performed an analysis in which we specifically looked at displacement over time by referencing each trajectory to its initial starting point. In this way, we measured changes in movement relative to an “internal” frame of reference.

Identification of NREM sleep epochs was performed by visual assessment of LFP during spontaneous recordings in 10-s increments (S6 Fig). During any period denoting sleep, if there was a sustained reduction >2 s in the amplitude of the slow-wave activity below threshold during a continuous epoch we excluded these segments. Neural activity from sleep epochs during spontaneous recordings was concatenated together, in order to analyze ensemble activations specifically during NREM sleep. All sleep-related analyses were constrained to the minimum amount of sleep achieved in either sleep epoch, in order to ensure that analyses were not biased by different amounts of sleep post-learning vs pre-learning.

### Neural Analysis

#### Peri-event task related neural analysis

Task-related peri-event time histograms were created for all units by time-locking unit activity (*t*_bin_ = 25 ms) to the onset of reach in each trial, as determined by frame-by-frame video analysis. For statistical analysis, smoothing of these PETH was performed using a Bayesian adaptive-regression spline algorithm [45,73,74], implemented within MATLAB using toolboxes downloaded at (http://www.cnbc.cmu.edu/~rkelly/code.html). B-spline regression algorithms have demonstrated clear benefits in estimating a fit around Poission distributed point-processes (i.e., spike-trains), compared to both Gaussian kernel density and logspline smoothing curves [45,74,75], particularly in cases in which there are dynamic changes in the rate of change of spike trains (i.e., both fast and slow changes in activity intensity) [73]. The algorithm automatically optimized for the number and location of “knots” (i.e., regions in which a new local regression model improves the overall fit of the curve) was determined automatically using a Markov chain Monte Carlo implemented to optimize the Bayes Information Criteria [45]. Modulation depth was calculated from these recordings based on the ratio of the peak amplitude to the base-line firing rate (baseline calculated by taking the mean firing-rate across a 250 ms time-period starting 1,500 ms prior to the onset of reach). Timing was estimated as the time from the onset of reach at which neurons reached peak-firing rate.

#### Ensemble activation analyses

To characterize ensemble reactivations following sleep, we performed an analysis that compared neural activity patterns during Sleep_1_ and Sleep_2_ with a template that was created during task execution in the awake period [29,48–50] (S6 Fig). We first computed a pairwise unit activity correlation matrix during the motor task by concatenating binned spike trains (*t*_bin_ = 25 ms) for each neuron across trials (500 ms prior to the onset of reach up to 1,000 ms after the onset of reach for each trial). This concatenated spike train was z-transformed, and then organized into a 2-D matrix organized by neurons (x) and time (B for number of time bins). From this spike count matrix, we calculated the correlation matrix (C_task_), and then calculated the eigenvector for the largest eigenvalue from this correlation matrix to study. This eigenvector was used as the ensemble template of activity, which was then projected back on to the neural activity trains from the same population of neurons during Sleep_1_ and Sleep_2_. This projection is a linear combination of Z-scored binned neural activity from the two blocks above, weighted by the PC ensemble (i.e., the eigenvector) calculated from the Reach task matrix. This linear combination has been described as the “activation strength” of that particular ensemble. In this analysis we focused on the first eigenvalue. Across the four animals, the first PC explained 13% of the variance; the second eigenvalue explained 7.6% of the variance and the third explained 6.5% of the variance.

#### Reactivation triggered LFP analysis

During sleep epochs, data was filtered (using a third order butterworth filter within MATLAB) across three different frequencies: 0.5–4 Hz (to capture slow wave oscillations); 9–12 Hz (to capture slow-spindles), and 13–16 Hz (to capture fast spindles). Next we concatenated data from 500 ms before and after ensemble reactivation events (in this case, using the top 10% of re-activations), and calculated the mean/standard error across time for each of these frequencies of interest. Statistical comparisons were performed using a repeated-measures ANOVA over time, followed by post-hoc *t* tests to identify specific time points that were significantly different between the two sleep sessions. We calculated the coefficient of variation (CV) to estimate the temporal coupling of the re-activation events with these oscillations. CV was calculated as the absolute value of the standard deviation (STD) divided by the absolute value of the mean across reactivation events for each time point. To compare changes in CV, we performed a paired *t* test across all time points showing significant mean differences in the overall amplitude between Sleep_1_ and Sleep_2_ sessions.

Intantaneous phase and amplitude of these spindle oscillations were calculated using a Hilbert transform of these event-triggered averages. Statistics on phase-changes were calculated using the MATLAB circular statistics toolbox [53].

#### Temporal sequence analysis

To assess for preserved relationship in temporal sequences, we used a template matching method described previously [51]. In hippocampal paradigms, neural activity that occurs during a trial is often used to define a task-template. Because of significantly greater trial-to-trial noise in the motor reach task, particularly during early learning sessions, we instead used the PETH across trials during Reach_1_ to define the “task-template.” To create the task-template (as shown across neurons in S3 Fig), we first binned neural activity at a 25 ms resolution starting 1,000 ms prior to the reach up to 1,500 mg after each reach. A PETH was created of this activity across trials, and this PETH was then z-scored. To create the task-template, this z-scored activity was further binned at various resolutions (at a resolution of 50 ms up to a resolution of 1,250 ms), producing a spike matrix with X-dimensions varying from 25 bins to 1 bin. This task-template was then compared with a Zscored matrix of spiking activity taken from each reactivation event at a 1 ms resolution (thus, a spike-template with 25 bins was compared with spiking activity across 25 ms; a template with 10 bins was compared with spiking activity across 10 ms, etc.). For each reactivation event, we produced a Pearson correlation-value determined as the greatest correlation between the two matrices (i.e., for the 10ms template, the template was moved along the 25 ms of the reactivation event, correlation was calculated at each location, and the maximum correlation value was kept for further analysis). Post-hoc testing of correlation values was performed post versus pre using Wilcox ranksum test.

### Automated Spindle Detection

Spindle detection was used using automated algorithms to detect such oscillations, adapting methods previously described [76]. Spindles were then detected using a threshold of 2.5 SD of the signal, with start and finish times calculated as the time points 1.5 SD of the signal. Events were identified as spindles only if they were longer than 400 ms and shorter than 3 s. Automatically detected spindles/delta-oscillations were visually inspected to ensure the algorithm was correctly detecting these events. We also tested more stringent criteria (3 SD of the signal for example); results reported here (an increase in spindle oscillations after learning) do not depend on the specific parameters chosen.

### Statistical analysis

We performed either non-parametric tests (Wilcox signrank/ranksum) or one–way ANOVA with post-hoc Fisher’s for most comparisons, as noted in the text. Logistic regression was used to identify changes in binary measures of success rate during learning or after sleep.

## Supporting Information

S1 Data Raw data (in excel format) for supplementary figures. (XLSX)

S2 Data Matlab-file for trajectories analyzed in S2 Fig. (MAT)

S3 Data Raw data (in excel format) for the main figures. (XLSX)

S1 Fig Placement of electrodes. Rodent motor cortex has been mapped out extensively over the preceding decades [30,31,32]. This motor mapping has demonstrated a consistent pattern in which there are two forelimb representations: a “caudal forelimb area” and a “rostral forelimb area,” typically separated by neck. Prior studies have demonstrated that caudal forelimb is associated with somatotopic reorganization following motor skill learning with, in particular, an increase in the representation of distal forelimb regions (i.e., wrist/digits) [30,31]. For these reasons, we chose coordinates within the part of caudal forelimb area most likely to represent these distal forelimb regions (3.5 mm lateral to bregma, 0.5 mm anterior to bregma) to center our probes **(A,B)**.
(TIF)

S2 Fig Change in kinematics during learning. In addition to calculating speed and accuracy, we also analyzed the mean movement trajectories during learning. **(A)** Using bins as described in Materials and Methods to define Reach_1early_ trials, Reach_1late_ trials, and Reach_2early_ trials, we calculated and plotted the mean X-Y coordinates for one second, or until the trajectory was finished, defined as when animal’s forepaw returned into the cage). In this example, the pellet is in a defined location (marked with the black dot), and trajectories are performed with the “external” frame of reference. We performed a statistical analysis of this state-space by calculating, at each X-position, the distribution of Y points associated with it. For each group, we calculated the distribution of Y-points associated with each X coordinate, and then used a bootstrap approach (i.e., using random sampling with replacement) to calculate confidence intervals at the *p* < 0.05 two-tailed significance level around the mean differences between groups. Using this analysis, we found significant changes in the starting and position of the trajectory (marked with * on figure. **(B)** We also calculated the “internally referenced” movement trajectory (i.e. referenced to each animal’s initial starting point), thus allowing for an analysis of movement independent of external reference points. Using the same bootstrapping approach to define confidence intervals, we again assessed for differences in the state-space trajectory. We found no significant changes during the online learning session, but significant changes in the trajectory after sleep (marked with *). Finally, because all internally referenced kinematics start from the same location, we were also able to calculate the angle at which these movements occurred. Using the maximum the max Euclidean distance from the starting coordinate to define the endpoint of each trajectory, we calculated the angle of each trajectory and used circular statistics to compute statistical change. We found no difference in the mean angle (mean angle of 135.2° for early trials and 136° for late trials in Reach_1_, *p* = 0.58), but a significant difference after sleep (mean trajectory angle of 140, *p* < 0.05 compared to Reach_1Late_). S1 Data and S2 Data contains raw trajectory analyses.
(TIF)

S3 Fig Distribution of single unit activity time-locked to reach onset. **(A)** Z-scored neural activity was plotted for all units, sorted by the time at which neurons reached peak firing rate (time = 0, reach onset), with data taken from the first reach block on day 1. Across the 102 neurons we recorded from four animals, we found a wide distribution of timings. However, there was a clear preponderance of neurons distributed to later portions of the reach task (for example 300 ms and onwards). This slightly skewed distribution is highly consistent with prior recordings from caudal forelimb region during forelimb reach tasks [46,77,78,79], and has been previously noted as different than primate cortical activity, which typically precedes the initial movement [46]. It has been speculated that in rodents earlier, pre-reach movements are predominately represented in rostral forelimb area [77] or in subcortical regions like red nucleus [78] or striatum [79]. In addition, prior evidence from intra-cortical mapping studies suggests there is a topographic representation of movements in forelimb motor cortex, with more medial regions of caudal forelimb area representing more proximal (elbow/shoulder) and “reach-related” movements, while lateral parts of caudal forelimb area represent more distal (i.e., wrist/digit) and “retract”-related movements [30,31,32]. Our electrode was placed primarily in regions of motor cortex thought to reflect distal forelimb (i.e., wrist/digit), and retract-related movements.
(TIF)

S4 Fig Online and offline change in timing of neural activity. We plotted the entire distribution of changes in neural timing during learning (**A)** and after sleep (**B)**. We show here that the offline changes in timing discussed in the main text occur across most neurons being studied, and “mean” changes in timing of neural activity after sleep reported in the text are not being skewed by a few outliers. See S1 Data for raw numbers.
(TIF)

S5 Fig Control extra-sleep experiment. **(A)** We performed a control experiment in which animals were allowed to sleep for two blocks before the skilled reach training to assess whether extra sleep prior to learning is sufficient for animals to improve and stabilize their kinematics. In addition, we gave animals up to 200 trials each day in order to assess if simply more training was sufficient to allow animals to improve their kinematic speed. Finally, we followed this with a 24-h period of rest to allow animals to sleep during their natural sleep period. This experiment demonstrated that extra sleep prior to learning and increased numbers of trials were not sufficient to produce significant changes in kinematic speed. **(B)** Example in one animal demonstrating lack of behavioral improvement until after sleep. **(C)** Quantification across animals demonstrates a lack of improvement in speed during the training session (*p* = 0.36), and significant decrement in reach-retract time after sleep (*p* < 0.0001). **(D)** As before, we found that accuracy improves during the initial reach training (*p* < 0.001), with retention but no further improvement in accuracy after sleep (*p* = 0.11). See S1 Data for raw numbers.
(TIF)

S6 Fig PCA method of detecting neural ensembles and assessing reactivation during sleep. **(A).** This method, adapted largely from [48–50] uses PCA to first detect task-related neural ensembles; and then assesses reactivation of these ensembles during sleep. To accomplish this, we first concatenated (binned and Z-scored) single-unit neural activity recorded during the reach task to produce a N × T matrix (where N represents the number of neurons recorded from, and T is the total time of the concatenated task-related data). In this study, we concatenated 1.5 seconds of neural activity from each trial. **(B)** We calculated the N × N correlation matrix from this N × T matrix in Matlab, and then extracted the top principle component (PC) from this matrix based on the ranges of eigenvalues. **(C)** The PC reflected common task-related variance (i.e., synchrony) across a set of neurons, and is represented as a series of “weights,” from -1 to 1, assigned to each neurons based on how much they contribute to the overall PC. Thus, those neurons with the highest weights were the dominant neurons in the ensemble; while neurons whose weights are close to 0 were not represented in that ensemble. Neurons with negative weights are inversely coupled to that particular ensemble. **(D)** To assess reactivation of this ensemble, we binned/Z-scored the N × S_T_ spike matrix (N represents number of neurons being recorded; S_T_ represents time during the sleep block), and multiplied this with the ensemble to produce a linear array of size 1 × S_T_ that represented the activity of that ensemble over the respective time period. In this way, we could assess the reactivation of the ensemble both during sleep and during awake states; before, during, and after the skilled reach task. In this analysis, we have focused on ensemble activity that occurred during sleep epochs either before or after the reach task.
(TIF)

S7 Fig Relationship of synchronous task-evoked activity with re-activation. **(A)** We first identified task-related neural ensembles by applying PCA to task-related neural activity (i.e., in order to generate principle component weights). Panel shows example of task-related activity in one animal. **(B)** Panel shows a typical example of the ordering of PC weights for the task-related firing. Color coding was used to indicate the top and the bottom half of weights. These PC weights were then convolved with the Z-scored spike matrix that occurred during subsequent sleep periods. **(C)** To understand the relationship of reactivations with single-unit spike data and the PC weights, we took the top ten percent of reactivation events and created a PETH for each neuron **binned at 25 ms**. At this course resolution, there was limited evidence of temporal jitter across neurons—i.e., all neurons showed some time-locking to these reactivation events. Colormap indicates the relative firing rate for the PETH (red = higher firing). Thus, the highest PC weights (e.g., units 1–10) resulted in the highest PETH firing during reactivation. Thus, there was a direct relationship between the PC weight and its summed activity in the reactivation, demonstrating one feature the reactivation is picking up is task-related variations in synchronous firing across a population of neurons and the degree to which this variation mimics the variation observed during the task. **(D)** We used a template match approach to assess whether there was temporal variation in neural firing at a micro-level. Task-related data across 1.25 s was binned at various resolutions (50 ms, 125 ms, 250 ms, and 1,250 ms) in order to assess for correlations with reactivation across 25 ms, 10 ms, 5 ms, and 1 ms respectively. Greater binning is associated with less temporal structure. We found generally that reactivation events showed greater correlation with task-related reactivation with more binning (i.e., with less temporal structure), suggesting that these reactivation events are associated more strongly with synchronous co-activation as opposed to temporal replay. **(E)** Examples of a reactivation event in Sleep_1_ task related activations and reactivation event in Sleep_2_.
(TIF)

S8 Fig Tetrode spike sorting. Tetrodes were sorted, as described in Materials and Methods, using a MATLAB-based toolbox (UltraMegaSort [72]). Sorting quality was estimated by visualizing ISI (inter-spike interval) for each sorted unit, cross-corellogram, PC space and the linear discriminant analysis, with all of these metrics used for deciding whether units are well sorted or are being over-sorted.
(TIF)

S9 Fig Identification of NREM sleep. Ten seconds of neural activity (i.e. LFP) and neck electromyography activity (trace is differential rectified activity recorded from two ball electrodes implanted into the neck muscle). NREM sleep was scored manually for all animals by assessing for large-amplitude slow-wave activity in 10-s increments. Only NREM sleep episodes lasting more than 1 min were included for the purposes of this analysis.
(TIF)

S1 Table Table of animals and probes used in experiment. (DOCX)

## Abbreviations

CV: coefficient of variation
ETA: event-triggered average
LFP: local field potential
LTP: Long-Term Potentiation
NREM: non-rapid eye movement
PC: principle component
PCA: principle components analysis
PETH: peri-event time histogram
