# Supplementary figures and images for: Sleep-Dependent Reactivation of Ensembles in Motor Cortex Promotes Skill Consolidation
Source: PLoS Biol. 2015 Sep 18;13(9):e1002263. doi: 10.1371/journal.pbio.1002263 (PMC4575076; doi:10.1371/journal.pbio.1002263)

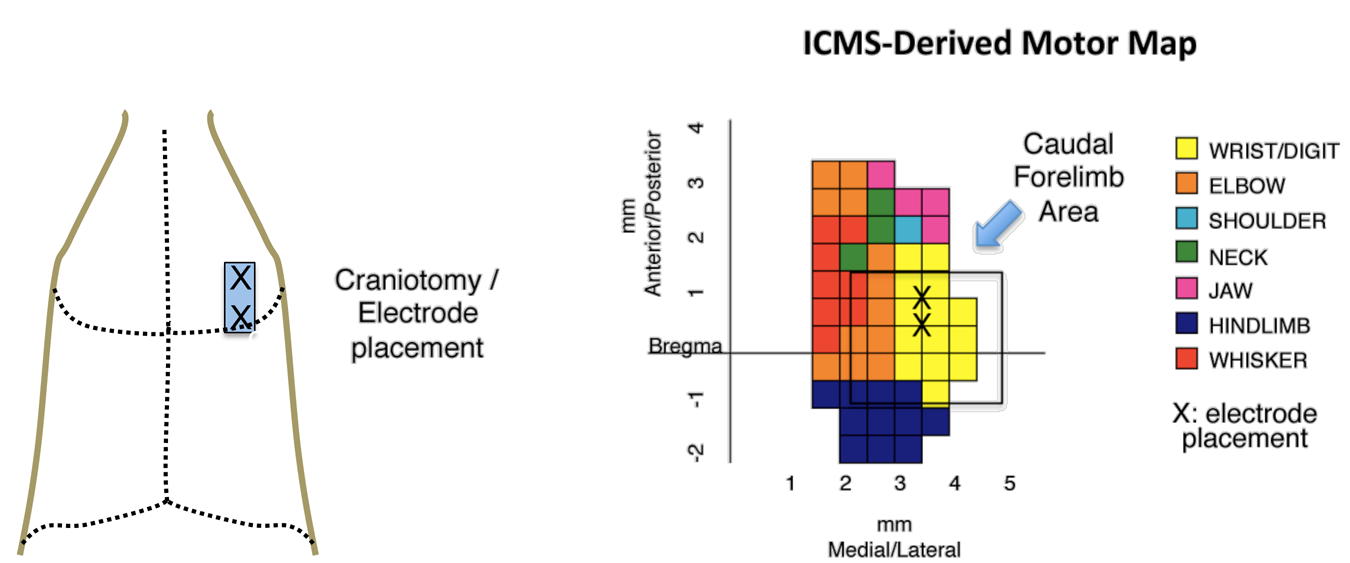

Supplement: S1 Fig — Rodent motor cortex has been mapped out extensively over the preceding decades [30,31,32]. This motor mapping has demonstrated a consistent pattern in which there are two forelimb representations: a “caudal forelimb area” and a “rostral forelimb area,” typically separated by neck. Prior studies have demonstrated that caudal forelimb is associated with somatotopic reorganization following motor skill learning with, in particular, an increase in the representation of distal forelimb regions (i.e., wrist/digits) [30,31]. For these reasons, we chose coordinates within the part of caudal forelimb area most likely to represent these distal forelimb regions (3.5 mm lateral to bregma, 0.5 mm anterior to bregma) to center our probes (A,B). (TIF) [file pbio.1002263.s004.tif]

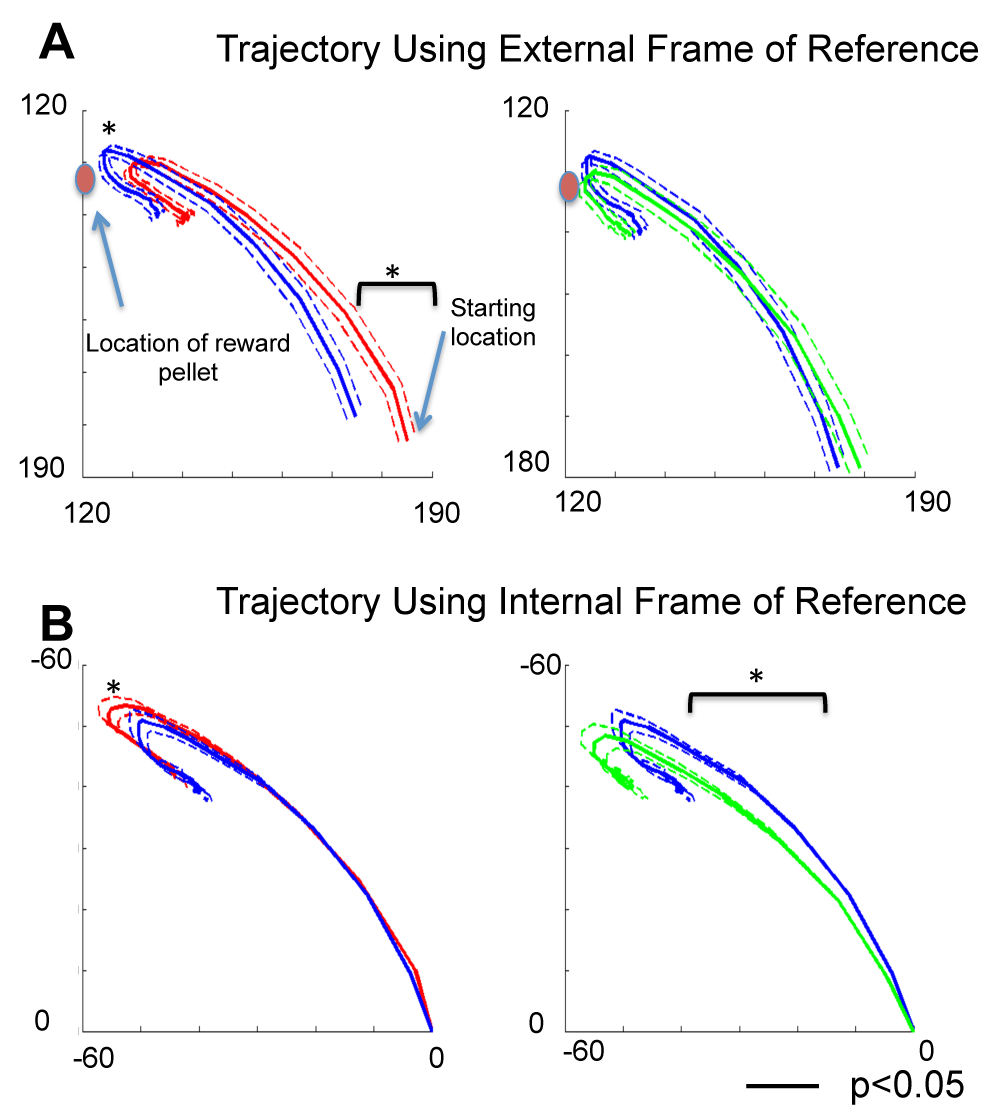

Supplement: S2 Fig — In addition to calculating speed and accuracy, we also analyzed the mean movement trajectories during learning. (A) Using bins as described in Materials and Methods to define Reach1early trials, Reach1late trials, and Reach2early trials, we calculated and plotted the mean X-Y coordinates for one second, or until the trajectory was finished, defined as when animal’s forepaw returned into the cage). In this example, the pellet is in a defined location (marked with the black dot), and trajectories are performed with the “external” frame of reference. We performed a statistical analysis of this state-space by calculating, at each X-position, the distribution of Y points associated with it. For each group, we calculated the distribution of Y-points associated with each X coordinate, and then used a bootstrap approach (i.e., using random sampling with replacement) to calculate confidence intervals at the p < 0.05 two-tailed significance level around the mean differences between groups. Using this analysis, we found significant changes in the starting and position of the trajectory (marked with * on figure. (B) We also calculated the “internally referenced” movement trajectory (i.e. referenced to each animal’s initial starting point), thus allowing for an analysis of movement independent of external reference points. Using the same bootstrapping approach to define confidence intervals, we again assessed for differences in the state-space trajectory. We found no significant changes during the online learning session, but significant changes in the trajectory after sleep (marked with *). Finally, because all internally referenced kinematics start from the same location, we were also able to calculate the angle at which these movements occurred. Using the maximum the max Euclidean distance from the starting coordinate to define the endpoint of each trajectory, we calculated the angle of each trajectory and used circular statistics to compute statistical change. We found no di [file pbio.1002263.s005.tif]

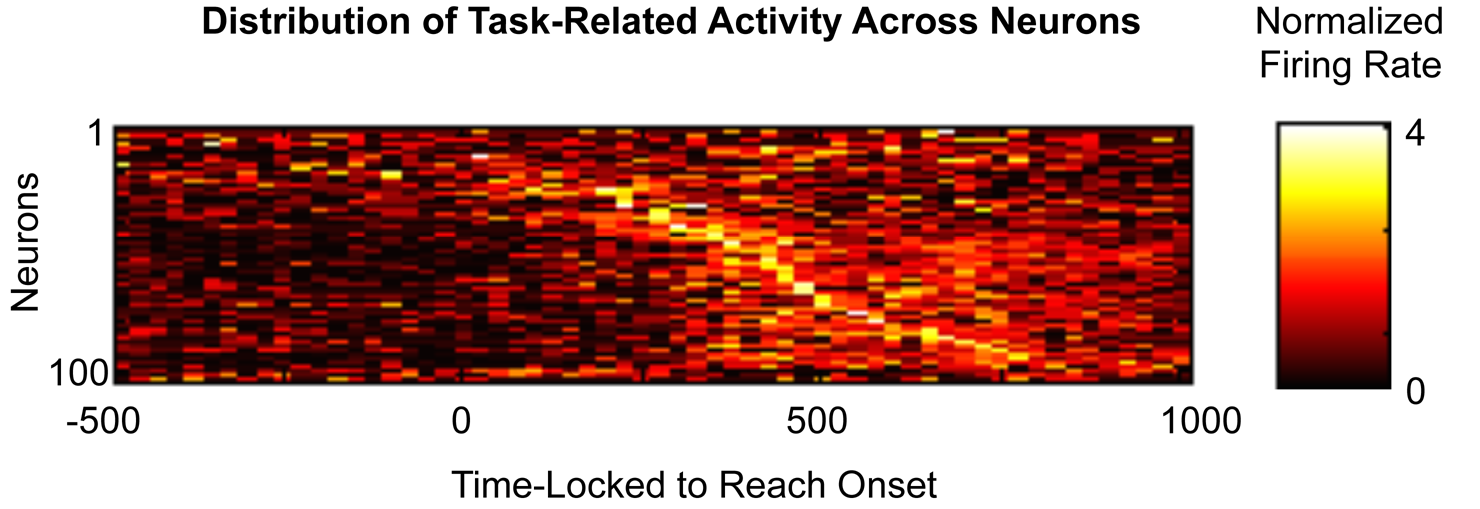

Supplement: S3 Fig — (A) Z-scored neural activity was plotted for all units, sorted by the time at which neurons reached peak firing rate (time = 0, reach onset), with data taken from the first reach block on day 1. Across the 102 neurons we recorded from four animals, we found a wide distribution of timings. However, there was a clear preponderance of neurons distributed to later portions of the reach task (for example 300 ms and onwards). This slightly skewed distribution is highly consistent with prior recordings from caudal forelimb region during forelimb reach tasks [46,77,78,79], and has been previously noted as different than primate cortical activity, which typically precedes the initial movement [46]. It has been speculated that in rodents earlier, pre-reach movements are predominately represented in rostral forelimb area [77] or in subcortical regions like red nucleus [78] or striatum [79]. In addition, prior evidence from intra-cortical mapping studies suggests there is a topographic representation of movements in forelimb motor cortex, with more medial regions of caudal forelimb area representing more proximal (elbow/shoulder) and “reach-related” movements, while lateral parts of caudal forelimb area represent more distal (i.e., wrist/digit) and “retract”-related movements [30,31,32]. Our electrode was placed primarily in regions of motor cortex thought to reflect distal forelimb (i.e., wrist/digit), and retract-related movements. (TIF) [file pbio.1002263.s006.tif]

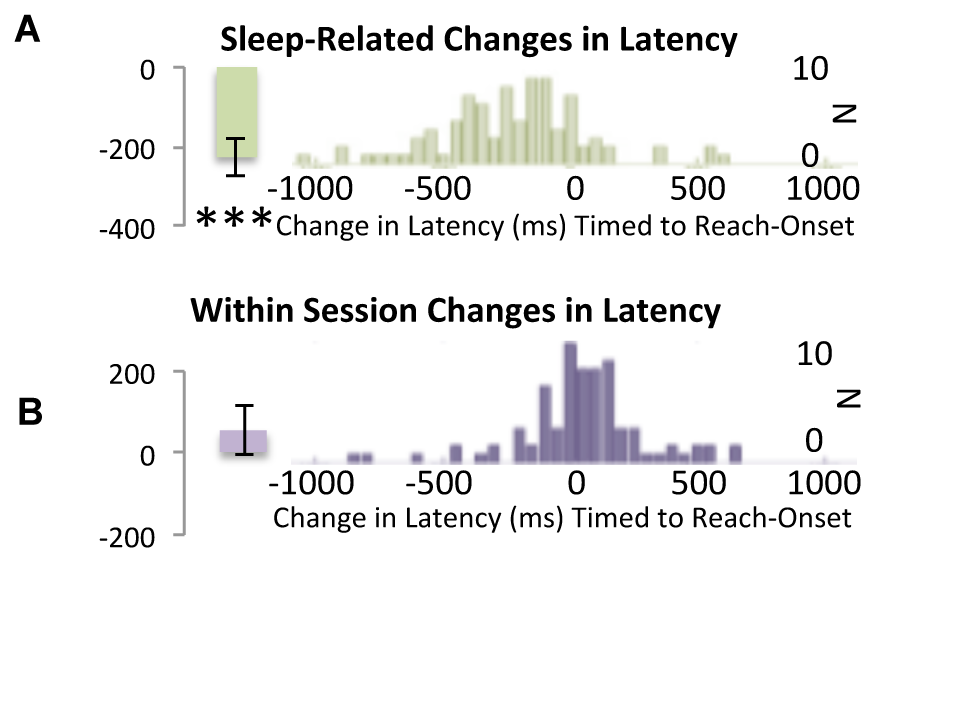

Supplement: S4 Fig — We plotted the entire distribution of changes in neural timing during learning (A) and after sleep (B). We show here that the offline changes in timing discussed in the main text occur across most neurons being studied, and “mean” changes in timing of neural activity after sleep reported in the text are not being skewed by a few outliers. See S1 Data for raw numbers. (TIF) [file pbio.1002263.s007.tif]

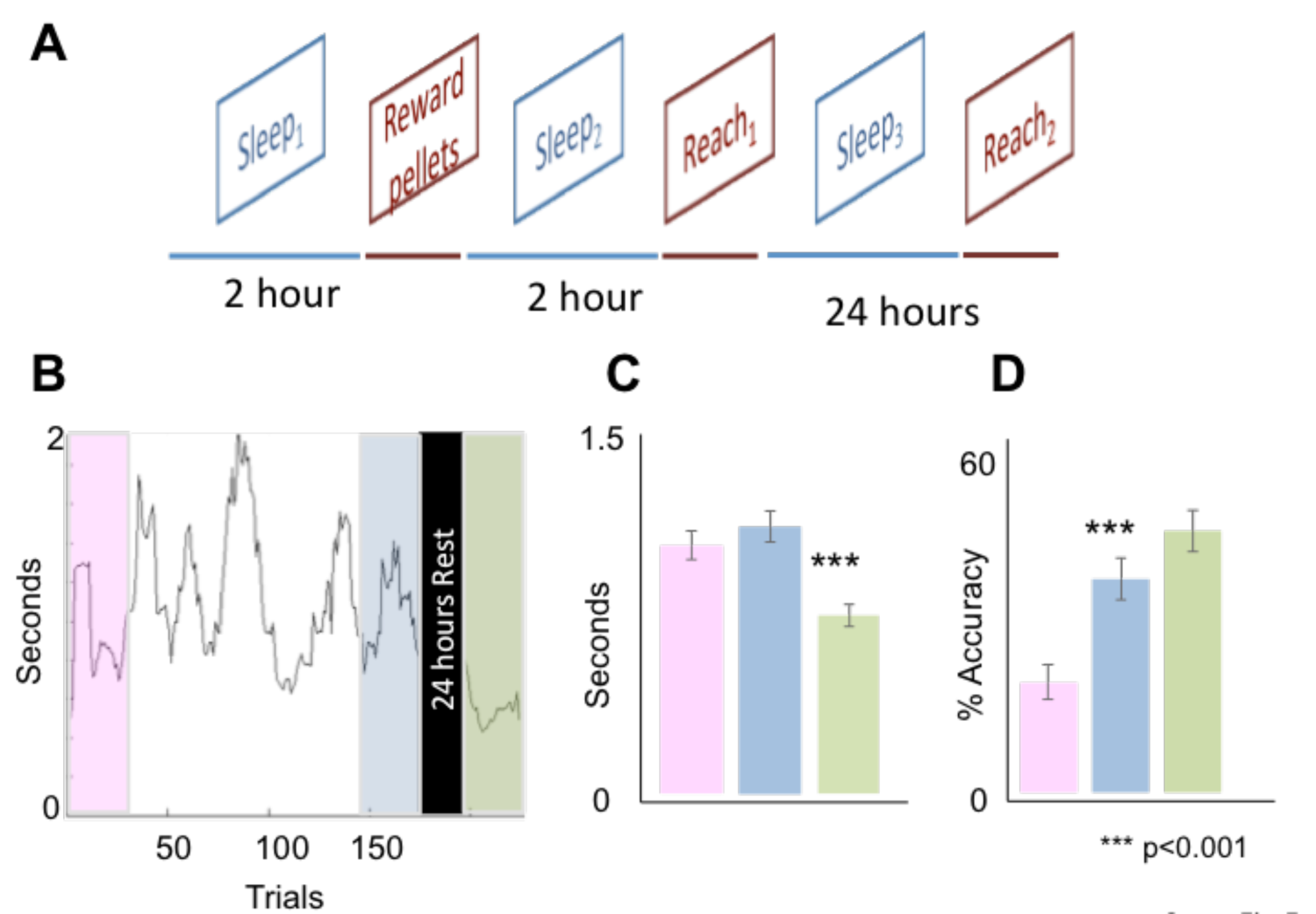

Supplement: S5 Fig — (A) We performed a control experiment in which animals were allowed to sleep for two blocks before the skilled reach training to assess whether extra sleep prior to learning is sufficient for animals to improve and stabilize their kinematics. In addition, we gave animals up to 200 trials each day in order to assess if simply more training was sufficient to allow animals to improve their kinematic speed. Finally, we followed this with a 24-h period of rest to allow animals to sleep during their natural sleep period. This experiment demonstrated that extra sleep prior to learning and increased numbers of trials were not sufficient to produce significant changes in kinematic speed. (B) Example in one animal demonstrating lack of behavioral improvement until after sleep. (C) Quantification across animals demonstrates a lack of improvement in speed during the training session (p = 0.36), and significant decrement in reach-retract time after sleep (p < 0.0001). (D) As before, we found that accuracy improves during the initial reach training (p < 0.001), with retention but no further improvement in accuracy after sleep (p = 0.11). See S1 Data for raw numbers. (TIF) [file pbio.1002263.s008.tif]

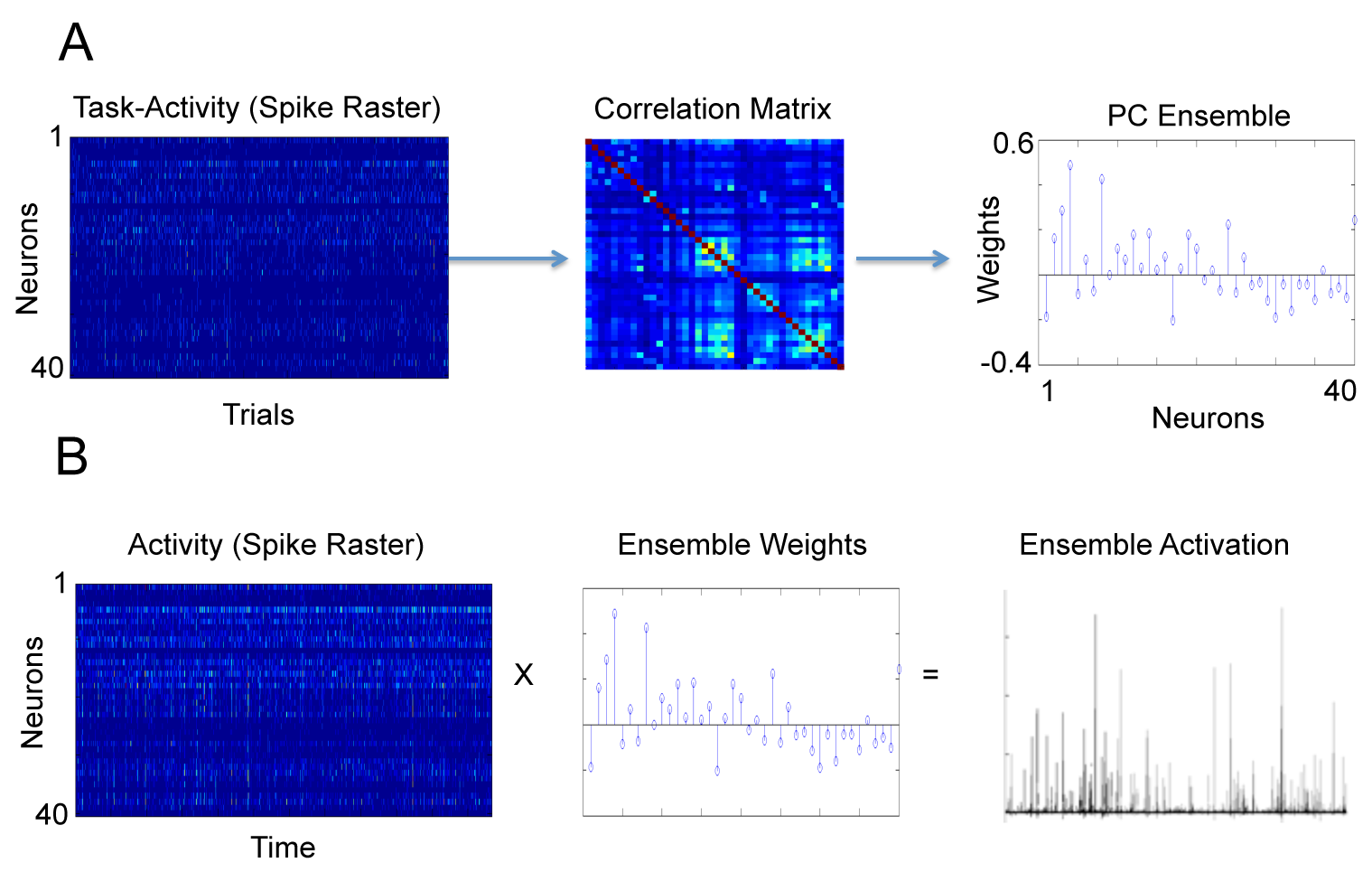

Supplement: S6 Fig — (A). This method, adapted largely from [48–50] uses PCA to first detect task-related neural ensembles; and then assesses reactivation of these ensembles during sleep. To accomplish this, we first concatenated (binned and Z-scored) single-unit neural activity recorded during the reach task to produce a N × T matrix (where N represents the number of neurons recorded from, and T is the total time of the concatenated task-related data). In this study, we concatenated 1.5 seconds of neural activity from each trial. (B) We calculated the N × N correlation matrix from this N × T matrix in Matlab, and then extracted the top principle component (PC) from this matrix based on the ranges of eigenvalues. (C) The PC reflected common task-related variance (i.e., synchrony) across a set of neurons, and is represented as a series of “weights,” from -1 to 1, assigned to each neurons based on how much they contribute to the overall PC. Thus, those neurons with the highest weights were the dominant neurons in the ensemble; while neurons whose weights are close to 0 were not represented in that ensemble. Neurons with negative weights are inversely coupled to that particular ensemble. (D) To assess reactivation of this ensemble, we binned/Z-scored the N × ST spike matrix (N represents number of neurons being recorded; ST represents time during the sleep block), and multiplied this with the ensemble to produce a linear array of size 1 × ST that represented the activity of that ensemble over the respective time period. In this way, we could assess the reactivation of the ensemble both during sleep and during awake states; before, during, and after the skilled reach task. In this analysis, we have focused on ensemble activity that occurred during sleep epochs either before or after the reach task. (TIF) [file pbio.1002263.s009.tif]

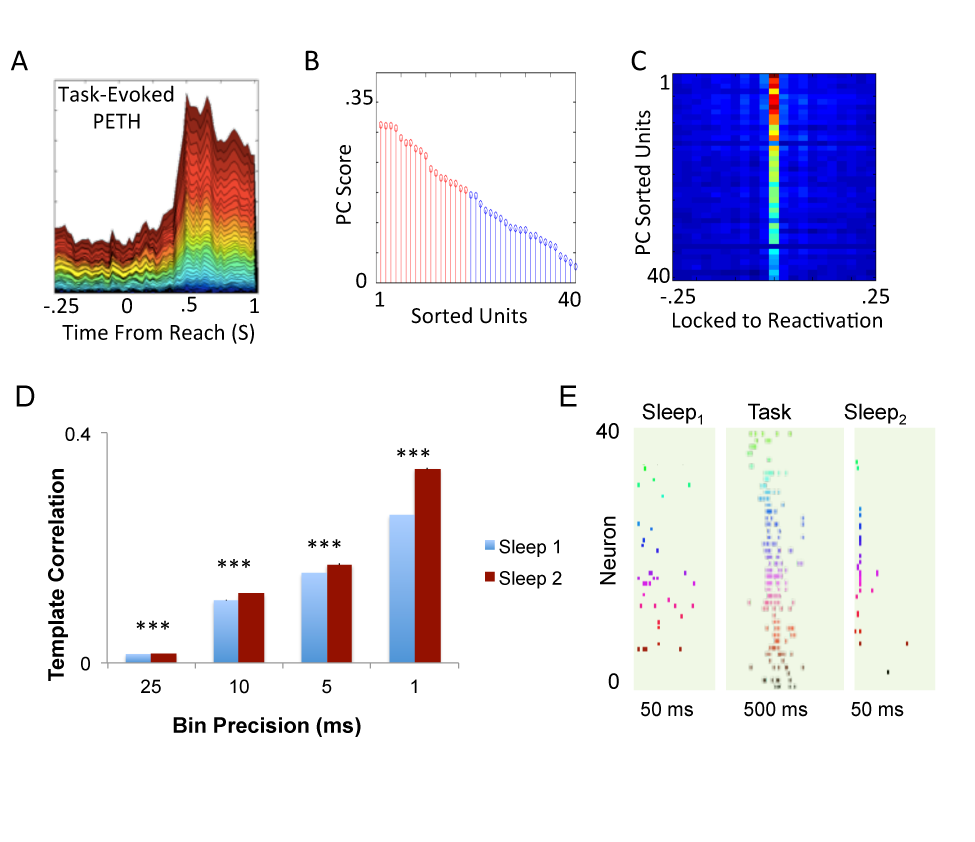

Supplement: S7 Fig — (A) We first identified task-related neural ensembles by applying PCA to task-related neural activity (i.e., in order to generate principle component weights). Panel shows example of task-related activity in one animal. (B) Panel shows a typical example of the ordering of PC weights for the task-related firing. Color coding was used to indicate the top and the bottom half of weights. These PC weights were then convolved with the Z-scored spike matrix that occurred during subsequent sleep periods. (C) To understand the relationship of reactivations with single-unit spike data and the PC weights, we took the top ten percent of reactivation events and created a PETH for each neuron binned at 25 ms. At this course resolution, there was limited evidence of temporal jitter across neurons—i.e., all neurons showed some time-locking to these reactivation events. Colormap indicates the relative firing rate for the PETH (red = higher firing). Thus, the highest PC weights (e.g., units 1–10) resulted in the highest PETH firing during reactivation. Thus, there was a direct relationship between the PC weight and its summed activity in the reactivation, demonstrating one feature the reactivation is picking up is task-related variations in synchronous firing across a population of neurons and the degree to which this variation mimics the variation observed during the task. (D) We used a template match approach to assess whether there was temporal variation in neural firing at a micro-level. Task-related data across 1.25 s was binned at various resolutions (50 ms, 125 ms, 250 ms, and 1,250 ms) in order to assess for correlations with reactivation across 25 ms, 10 ms, 5 ms, and 1 ms respectively. Greater binning is associated with less temporal structure. We found generally that reactivation events showed greater correlation with task-related reactivation with more binning (i.e., with less temporal structure), suggesting that these reactivation events are associated more strongly with [file pbio.1002263.s010.tif]

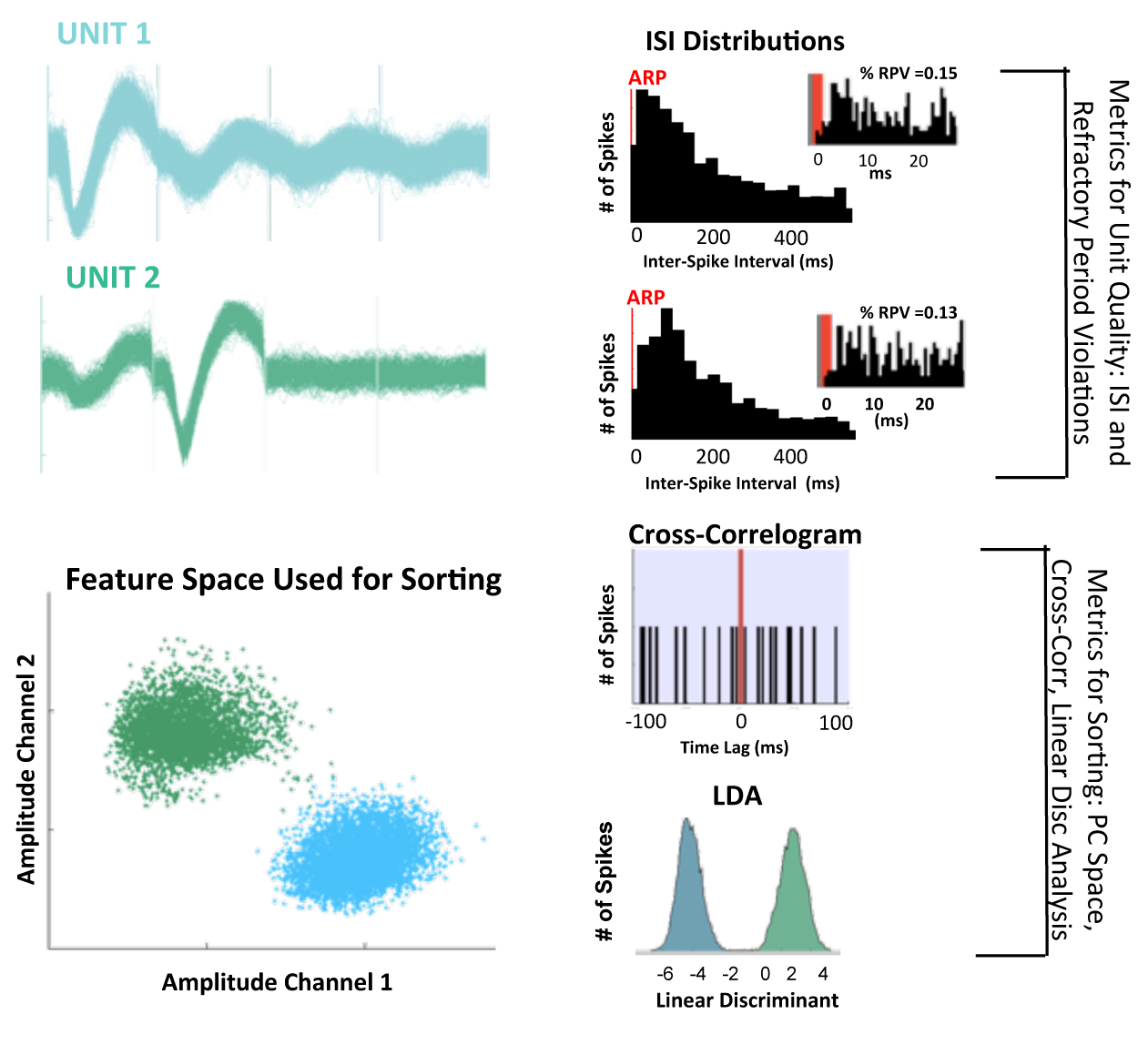

Supplement: S8 Fig — Tetrodes were sorted, as described in Materials and Methods, using a MATLAB-based toolbox (UltraMegaSort [72]). Sorting quality was estimated by visualizing ISI (inter-spike interval) for each sorted unit, cross-corellogram, PC space and the linear discriminant analysis, with all of these metrics used for deciding whether units are well sorted or are being over-sorted. (TIF) [file pbio.1002263.s011.tif]

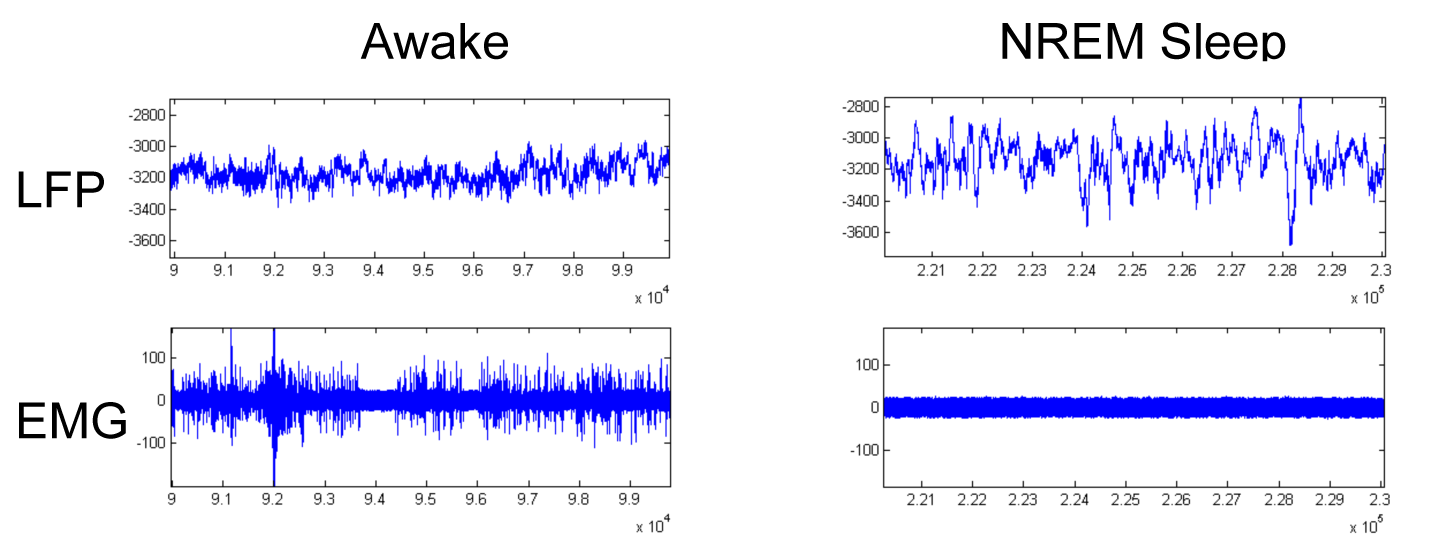

Supplement: S9 Fig — Ten seconds of neural activity (i.e. LFP) and neck electromyography activity (trace is differential rectified activity recorded from two ball electrodes implanted into the neck muscle). NREM sleep was scored manually for all animals by assessing for large-amplitude slow-wave activity in 10-s increments. Only NREM sleep episodes lasting more than 1 min were included for the purposes of this analysis. (TIF) [file pbio.1002263.s012.tif]
