# Supplementary material for: Sleep-Dependent Reactivation of Ensembles in Motor Cortex Promotes Skill Consolidation
Source: PLoS Biol. 2015 Sep 18;13(9):e1002263. doi: 10.1371/journal.pbio.1002263 (PMC4575076; doi:10.1371/journal.pbio.1002263)
Supplement: S1 Table — (DOCX) [file pbio.1002263.s013.docx]

**SUPPLEMENTARY TABLE 1**

|  |  |  | |  |
| --- | --- | --- | --- | --- |
| Animals | Probe | # units | Experiment | |
| 1 | microwire | 11 | sleep | |
| 2 | tetrode | 40 | sleep | |
| 3 | microwire | 38 | sleep | |
| 4 | tetrode | 13 | sleep | |
| 5 | microwire | LFP only,no units | sleep | |
| 6 | tetrode | 12 | sleep-restriction | |
| 7 | microwire | 68 | sleep-restriction | |
| 8 | microwire | LFP only, no units | sleep-restriction | |
| 9 | -- |  | sleep-restriction | |
| 10 | -- |  | sleep restriction | |
| 11 | -- |  | Extra-sleep control | |
| 12 | -- |  | Extra-sleep control | |
| 13 | -- |  | Extra-sleep control | |
| 14 | -- |  | Extra-sleep control | |
| 15 | -- |  | Extra-sleep control | |
